# A Kinase-Independent Role for the Rad3^ATR^-Rad26^ATRIP^ Complex in Recruitment of Tel1^ATM^ to Telomeres in Fission Yeast

**DOI:** 10.1371/journal.pgen.1000839

**Published:** 2010-02-05

**Authors:** Lakxmi Subramanian, Toru M. Nakamura

**Affiliations:** Department of Biochemistry and Molecular Genetics, University of Illinois at Chicago, Chicago, Illinois, United States of America; The University of North Carolina at Chapel Hill, United States of America

## Abstract

ATM and ATR are two redundant checkpoint kinases essential for the stable maintenance of telomeres in eukaryotes. Previous studies have established that MRN (Mre11-Rad50-Nbs1) and ATRIP (ATR Interacting Protein) interact with ATM and ATR, respectively, and recruit their partner kinases to sites of DNA damage. Here, we investigated how Tel1^ATM^ and Rad3^ATR^ recruitment to telomeres is regulated in fission yeast. Quantitative chromatin immunoprecipitation (ChIP) assays unexpectedly revealed that the MRN complex could also contribute to the recruitment of Tel1^ATM^ to telomeres independently of the previously established Nbs1 C-terminal Tel1^ATM^ interaction domain. Recruitment of Tel1^ATM^ to telomeres in *nbs1-c60Δ* cells, which lack the C-terminal 60 amino acid Tel1^ATM^ interaction domain of Nbs1, was dependent on Rad3^ATR^-Rad26^ATRIP^, but the kinase domain of Rad3^ATR^ was dispensable. Thus, our results establish that the Rad3^ATR^-Rad26^ATRIP^ complex contributes to the recruitment of Tel1^ATM^ independently of Rad3^ATR^ kinase activity, by a mechanism redundant with the Tel1^ATM^ interaction domain of Nbs1. Furthermore, we found that the N-terminus of Nbs1 contributes to the recruitment of Rad3^ATR^-Rad26^ATRIP^ to telomeres. In response to replication stress, mammalian ATR–ATRIP also contributes to ATM activation by a mechanism that is dependent on the MRN complex but independent of the C-terminal ATM interaction domain of Nbs1. Since telomere protection and DNA damage response mechanisms are very well conserved between fission yeast and mammalian cells, mammalian ATR–ATRIP may also contribute to the recruitment of ATM to telomeres and to sites of DNA damage independently of ATR kinase activity.

## Author Summary

ATM and ATR kinases are two evolutionarily conserved sensors of DNA damage, responsible for maintaining stable genomes in all eukaryotic cells. These two kinases safeguard eukaryotic genomes against undesired double-stranded DNA breaks (DSBs) and errors during duplication of genomic DNA. Furthermore, ATM and ATR are redundantly required for stable maintenance of telomeres, protective structures at ends of linear eukaryotic chromosomes. Our current study in fission yeast demonstrates that the previously defined C-terminal Tel1^ATM^ interaction domain of the DNA repair protein Nbs1, which contributes to recruitment of Tel1^ATM^ to DSBs, is dispensable for recruitment of Tel1^ATM^ to telomeres, due to a previously unrecognized kinase-independent role of ATR in recruitment of Tel1^ATM^ to telomeres. Furthermore, the N-terminus of Nbs1 was found to be critical for recruitment of both ATR and ATM to telomeres. Regulators of telomere maintenance have recently emerged as potentially important therapeutic targets against tumorigenesis and aging in mammalian cells. Since proteins responsible for proper maintenance of telomeres and cellular responses to DNA damage are highly conserved between fission yeast and mammalian cells, a newly uncovered molecular crosstalk between ATM and ATR might also play critical roles in telomere maintenance and DNA damage responses in mammalian cells.

## Introduction

ATM (Ataxia Telangiectasia Mutated) and ATR (ATM and Rad3-related), members of the phosphoinositol-3-kinase like kinase (PIKK) family, are central players in coordinating cellular responses to various forms of DNA damage, such as DNA double-stranded breaks (DSBs) and problems that are encountered by DNA replication forks, in eukaryotic cells [1],[2]. ATM and ATR both preferentially recognize and phosphorylate Serine (S) or Threonine (T) amino acid residues followed by Glutamate (Q), and over 900 sites in more than 700 proteins have been identified as potential phosphorylation sites for these two kinases in mammalian cells [3].

Previous studies have identified the Mre11-Rad50-Nbs1 (MRN) DNA repair complex as a key player in the activation of ATM kinase in response to DSBs [1],[4]. The MRN complex interacts with ATM through an evolutionarily conserved C-terminal motif in its Nbs1 subunit, and this interaction is critical for recruitment of ATM to DSBs and phosphorylation of downstream targets by ATM [5],[6]. Likewise, ATRIP (ATR-Interacting Protein) interacts with ATR through its evolutionarily conserved extreme C-terminal motif and promotes recruitment of ATR to sites of DNA damage [6]. RPA (Replication Protein A)-coated single-stranded DNA (ssDNA) serves as a platform for recruitment of the ATR-ATRIP complex, where phosphorylation of various downstream targets can take place [7]. Besides its role in activation of ATM, the MRN complex has also been shown to contribute to ATR signaling in mammalian cells [8].

Initial studies have suggested that ATM is particularly important for recognition of DSBs, while ATR is more important for recognition of replication stress and ultraviolet radiation (UV)-induced DNA damage [1],[2]. However, recent studies have uncovered an intimate crosstalk between the ATM and ATR signaling pathways in response to DNA damage. For example, ATM-MRN can act upstream of ATR-ATRIP in cellular responses to DSBs by promoting recruitment of ATR-ATRIP through its contribution to generate RPA-coated ssDNA at DSBs [9]–[11]. Conversely, ATR contributes to the activation of ATM in response to DNA replication stress by converting inactive ATM dimers into active monomers through direct phosphorylation of ATM [12]. However, it is currently unknown if ATR-ATRIP may also contribute to the recruitment of ATM to stalled replication forks. In contrast to DSB-induced ATM activation, the C-terminal ATM interaction domain of Nbs1 is not required for ATR-dependent activation of ATM in response to DNA replication stress; however, the Nbs1 N-terminus is essential for ATR-dependent activation of ATM [12].

**Figure 1:**
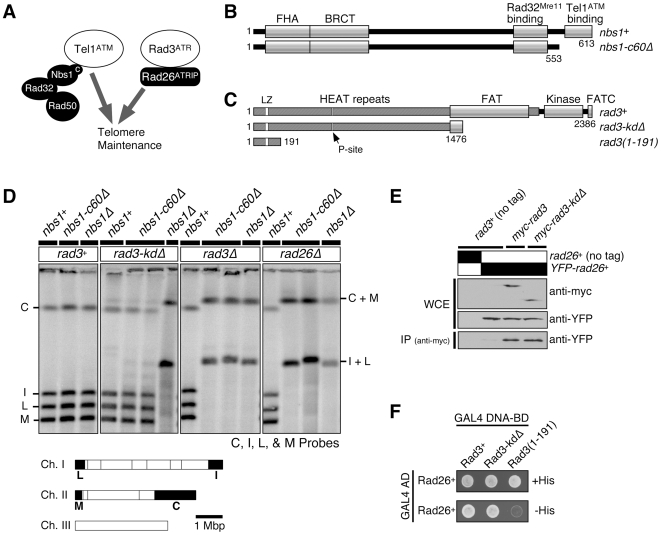
Kinase-independent function of Rad3^ATR^-Rad26^ATRIP^ contributes to telomere maintenance in the absence of the Nbs1-Tel1^ATM^ interaction domain of Nbs1. (A) Tel1^ATM^-MRN (Rad32^Mre11^-Rad50-Nbs1) and Rad3^ATR^-Rad26^ATRIP^ represent two redundant pathways essential for telomere maintenance in fission yeast. (B) Schematic representation of Nbs1 protein expressed in *nbs1^+^* and *nbs1-c60Δ*. Conserved motifs and functional domains [5],[27] are indicated. (C) Schematic representation of various Rad3 constructs tested. Amino acids 1477–2386 are deleted in *rad3-kdΔ*, while amino acids 1–2363 are deleted in *rad3Δ*. Conserved motifs and functional domains are indicated [26],[58]. The Leucine zipper (LZ) and putative PCNA-interaction site (P-site) were previously implicated in protein-protein interaction [59]. (D) Characterization of telomere status by pulsed-field gel electrophoresis (PFGE) for indicated strains. Cells with defects in telomere maintenance show C+M and I+L bands, corresponding to the fused telomeric fragments. Bottom, NotI restriction map of fission yeast chromosomes, shown with telomeric C, I, L, and M fragments marked as black boxes. (E) Rad3-kdΔ forms a stable complex with Rad26 *in vivo*. Anti-myc immunoprecipitation was performed using extracts from strains expressing YFP-Rad26 and either wild-type myc-Rad3 or myc-Rad3-kdΔ, and co-immunoprecipitated Rad26 was visualized by anti-GFP western blot. (F) Two-hybrid assays of *S. pombe* Rad26 with full-length Rad3 (Rad3^+^), C-terminal truncation of Rad3 (Rad3-kdΔ), or the N-terminal 191 amino acids of Rad3. A positive two-hybrid interaction is identified by growth on -His plates.

ATM-MRN and ATR-ATRIP are also redundantly required for the maintenance of telomeres, stable DSBs at ends of chromosomes, in a wide variety of eukaryotic species [13] (Figure 1A). Studies in yeasts and mammalian cells have shown that ATM-MRN and ATR-ATRIP are recruited to functional telomeres during S/G_2_ phases of the cell cycle [14]–[16]. In budding yeast, Tel1^ATM^ and Mec1^ATR^ have been shown to phosphorylate several Serine residues within the telomerase-recruitment domain of the telomere capping protein Cdc13, and these phosphorylation events have been proposed to promote efficient telomerase recruitment to telomeres [14],[17]. In fission yeast, we have recently shown that Tel1^ATM^ and Rad3^ATR^ redundantly promote interaction between the Pot1 telomere capping complex (consisting of Pot1, Tpz1, Poz1 and Ccq1 subunits) and telomerase, and thereby help to recruit telomerase to telomeres [18]. On the other hand, the telomeric GT-rich repeat DNA binding factors TRF2 and POT1, essential for protection of telomeres against degradation and recombination, play critical roles in attenuating DNA damage checkpoint activation mediated by ATM and ATR in mammalian cells [19]–[21]. Therefore, it has been suggested that telomeres transiently become de-protected during S- and G_2_-phases, and can thus be recognized as DSBs by ATM/ATR to allow the timely recruitment of telomerase [22].

On the other hand, we have recently found that the arrival of lagging strand DNA polymerases (α and δ) to telomeres is significantly delayed compared to the arrival of the leading strand DNA polymerase ε in fission yeast, and that significant quantities of RPA and Rad26^ATRIP^ transiently accumulate at replicating telomeres [15]. These observations thus raised the possibility that replicating telomeres may be primarily recognized as unusual/stressed replication forks by Rad3^ATR^-Rad26^ATRIP^ rather than being recognized as DSBs in fission yeast [15]. In mammalian cells, recruitment of ATR and MRN to telomeres precedes recruitment of ATM to telomeres [16], and replication of lagging-strand telomeres also appears to be significantly delayed compared to leading-strand telomeres [23]. Thus, replicating mammalian telomeres might also accumulate unusually high levels of RPA on the lagging strand and activate ATR, and ATR might subsequently contribute to the activation of ATM signaling at telomeres to promote stable telomere maintenance.

Here, we investigated inter-dependencies among Tel1^ATM^, Nbs1, and Rad3^ATR^-Rad26^ATRIP^ for their recruitment to telomeres in fission yeast. Our studies uncovered a surprising kinase-independent role of the Rad3^ATR^-Rad26^ATRIP^ complex, which is redundant with the C-terminal Tel1^ATM^-interacting domain of Nbs1, for the recruitment of Tel1^ATM^ to telomeres. We also demonstrate that the N-terminal domain of Nbs1 contributes to Rad3^ATR^-Rad26^ATRIP^ function by promoting recruitment of Rad3^ATR^-Rad26^ATRIP^ to telomeres. Thus, our findings provide mechanistic insights into how recruitment of Tel1^ATM^ and Rad3^ATR^ to telomeres is regulated, and define a novel molecular crosstalk between Rad3^ATR^ and Tel1^ATM^ in recognition of fission yeast telomeres, potentially resembling the ATR-ATM crosstalk in response to DNA replication stress in mammalian cells.

## Results

### Tel1^ATM^ interaction domain of Nbs1 is dispensable for telomere maintenance in *rad3-kdΔ* cells

The C-terminus of Nbs1 is important for its interaction with Tel1^ATM^ in fission yeast *Schizosaccharomyces pombe* [5] (Figure 1B). This interaction is evolutionarily conserved, since the corresponding region of Nbs1 is also required for its interaction with ATM in *Xenopus* and humans [5],[6]. Mutating or truncating the Nbs1 C-terminus in fission yeast cells lacking functional Rad3^ATR^ eliminated DNA damage-induced histone H2A phosphorylation (γ-H2A) and foci formation of the checkpoint mediator protein Crb2/Rhp9 [5], since Tel1^ATM^ and Rad3^ATR^ are redundantly required for DNA damage-induced histone H2A phosphorylation and Crb2 foci formation [24]. Tel1^ATM^-MRN (Rad32^Mre11^-Rad50-Nbs1) and Rad3^ATR^-Rad26^ATRIP^ pathways are also redundantly required for stable telomere maintenance, and simultaneous inactivation of these two pathways results in chromosome circularization in fission yeast [25] (Figure 1A). Thus, it was previously hypothesized that disruption of Tel1^ATM^-Nbs1 interaction in fission yeast cells lacking functional Rad3^ATR^ would lead to telomere dysfunction and chromosome circularization [5].

We tested this hypothesis by combining the most commonly used *rad3* deletion allele (*rad3Δ::ura4* ^+^, hereafter referred to as *rad3-kdΔ*) with the Nbs1 C-terminal 60 amino acid deletion allele *nbs1-c60Δ* that disrupts Tel1^ATM^-Nbs1 interaction [5],[26] (Figure 1B and 1C). After extensive restreaking of fission yeast cells on agar plates, we examined the status of telomeres by separating NotI-digested chromosomal DNA by pulsed-field gel electrophoresis (PFGE), and then performing Southern blot analysis with telomeric NotI fragment-specific probes C, I, L and M (Figure 1D). In this assay, cells carrying circular chromosomes lose individual telomeric NotI fragments and show I+L and C+M bands, corresponding to the fused telomeric fragments from chromosomes I and II.

We were surprised to find that the double mutant *nbs1-c60Δ rad3-kdΔ* cells stably maintained telomeres (Figure 1D). By contrast, combining *tel1Δ* or *nbs1Δ* with *rad3-kdΔ* caused complete loss of telomeres and chromosome circularization, as previously shown [25],[27] (Figure 1D). Thus, while Nbs1 and Tel1^ATM^ are both essential for telomere maintenance in *rad3-kdΔ* cells, the Nbs1-Tel1^ATM^ interaction is dispensable for telomere maintenance in *rad3-kdΔ* cells. The mutant Nbs1-c60Δ protein retains its ability to interact with Rad32^Mre11^, and both *rad32Δ rad3-kdΔ* and *rad50Δ rad3-kdΔ* cells are unable to maintain telomeres [5],[27]. Thus, the N-terminus of Nbs1 is required for the MRN (Rad32^Mre11^-Rad50-Nbs1) complex to fulfill its essential telomere maintenance function(s) in *rad3-kdΔ* cells.

### The N-terminal non-kinase domain of Rad3^ATR^ and its regulatory subunit Rad26^ATRIP^ are both required for telomere maintenance in the absence of Nbs1-Tel1^ATM^ interaction domain

The *rad3-kdΔ* allele does not completely delete the *rad3^+^* coding region and only removes a C-terminal portion of the Rad3^ATR^ protein including its kinase domain [26] (Figure 1C). We found that the truncated Rad3^ATR^ protein (Rad3-kdΔ^ATR^) expressed stably, and retained its ability to interact with its regulatory subunit Rad26^ATRIP^ in co-immunoprecipitation (co-IP) experiments and yeast 2-hybrid assays (Figure 1E and 1F). Similarly, the N-terminal regions of mammalian ATR and budding yeast Mec1, outside of their C-terminal kinase domains, interact with their respective regulatory subunits ATRIP and Ddc2 [28],[29]. Furthermore, when the fission yeast *rad3^+^* gene was originally cloned, a truncated Rad3^ATR^ protein lacking its kinase domain was found to fully suppress UV, IR and HU hypersensitivities and a G_2_-checkpoint defect of *rad3-136* cells [30]. In addition, over-expression of the N-terminal fragments of *Xenopus* ATR or budding yeast Mec1^ATR^ lacking the kinase domain has been shown to partially suppress the DNA damage sensitivity of *mec1-1* budding yeast cells by activating the spindle checkpoint [31]. Therefore, we next examined if a complete deletion allele of *rad3* ^+^ (*rad3Δ::LEU2*, hereafter referred to as *rad3Δ*, deletes genomic DNA corresponding to amino acids 1-2363 of Rad3^ATR^ [25]) would show a different genetic interaction with *nbs1-c60Δ* with respect to DNA damage sensitivity and telomere maintenance, even though we saw no difference between *rad3-kdΔ* and *rad3Δ* cells for their average telomere lengths in *nbs1^+^* background (Figure S1A).

While we detected no differences in sensitivity towards various DNA damaging agents between *rad3-kdΔ* and *rad3Δ* alleles in *nbs1* ^+^, *nbs1-c60Δ* or *nbs1Δ* backgrounds (Figure S1), we found that *rad3Δ nbs1-c60Δ* cells cannot maintain stable telomeres, unlike *rad3-kdΔ nbs1-c60Δ* cells (Figure 1D). Thus, the N-terminal domain of Rad3^ATR^ contributes to Tel1^ATM^-dependent telomere maintenance in *nbs1-c60Δ* cells. Additionally, we found that *rad26Δ nbs1-c60Δ* cells cannot maintain telomeres (Figure 1D), establishing that Rad26^ATRIP^ is essential for telomere maintenance in *nbs1-c60Δ* cells. Thus, these results indicated that the Rad3-kdΔ^ATR^-Rad26^ATRIP^ complex has a kinase-independent function for telomere maintenance in cells lacking the C-terminal Tel1^ATM^ interaction domain of Nbs1. The apparent discrepancy in absolute requirement for the Rad3^ATR^ kinase domain for DNA damage sensitivity versus telomere maintenance is plausible, since previous studies have established that the downstream checkpoint kinases Chk1 and Cds1^CHK2^ are not involved in telomere maintenance in fission yeast, while these kinases play critical roles in cell survival in response to DNA damage [25],[32].

### The N-terminal domain of Rad3^ATR^ is required for recruitment of Rad26^ATRIP^ to telomeres

**Figure 2:**
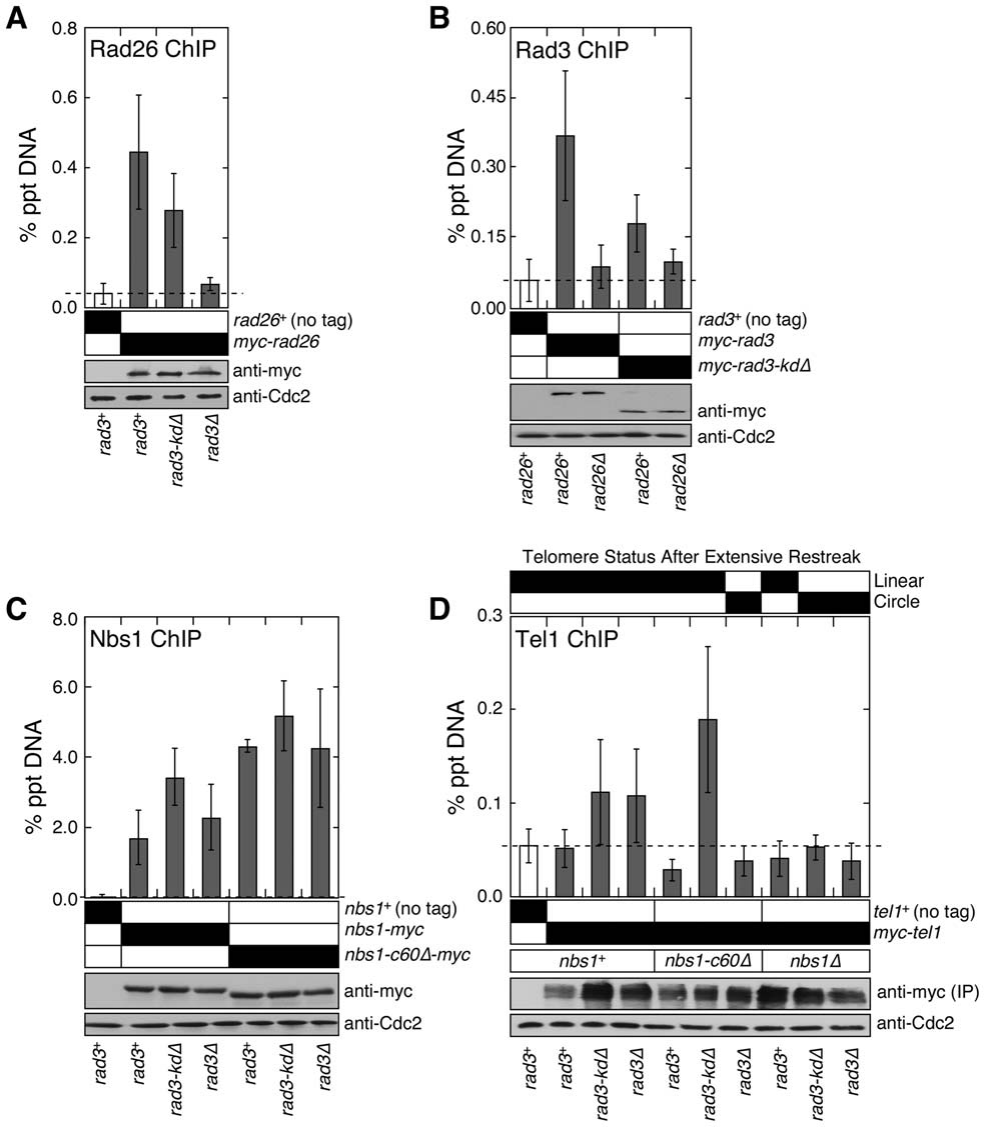
The C-terminal Tel1^ATM^ interaction domain of Nbs1 and the non-kinase domain of Rad3^ATR^ redundantly contribute to recruitment of Tel1^ATM^ to telomeres. (A) Recruitment of Rad26^ATRIP^ to telomeres in *rad3^+^*, *rad3-kdΔ*, and *rad3Δ*, monitored by quantitative ChIP assays. Mean values plus or minus one standard deviation for two to nine independent experiments are plotted. Compared to untagged control, Rad26^ATRIP^ showed significant telomere binding in *rad3^+^* and *rad3-kdΔ* (P values≤0.00003), but not in *rad3Δ* (P = 0.282). Based on anti-myc western blot, comparable level of Rad26 was expressed. Anti-Cdc2 western blots were used as loading controls. (B) Recruitment of wild-type Rad3^ATR^ or Rad3-kdΔ to telomeres in *rad26^+^* or *rad26Δ*, monitored by quantitative ChIP assays. Mean values plus or minus one standard deviation for three to eleven independent experiments are plotted. Compared to untagged control, both wild-type Rad3 and Rad3-kdΔ showed significant telomere binding in *rad26^+^* (P values≤0.0003) but not in *rad26Δ* (P values≥0.154). Based on anti-myc western blot, expression levels of wild-type or truncated Rad3^ATR^ are not affected by deletion of *rad26*. Anti-Cdc2 western blots were used as loading controls. (C) Recruitment of wild-type Nbs1 or Nbs1-c60Δ to telomeres in *rad3^+^*, *rad3-kdΔ*, or *rad3Δ*, monitored by quantitative ChIP assays. Mean values plus or minus one standard deviation for three to seven independent experiments are plotted. Compared to untagged control, both wild-type Nbs1 and Nbs1-c60Δ showed significant telomere binding in all genetic backgrounds tested (P values≤0.0001). Telomere binding of wild-type Nbs1 was significantly increased in *rad3-kdΔ* (P = 0.004) but not *rad3Δ* cells (P = 0.295), compared to *rad3^+^* cells. For Nbs1-c60Δ, there was no significant change in telomere association in *rad3-kdΔ* (P = 0.214) or *rad3Δ* cells (P = 0.952), compared to *rad3^+^* cells. Based on anti-myc western blot, expression levels of wild-type or truncated Nbs1 are not affected by mutations in Rad3. Anti-Cdc2 western blots were used as loading controls. (D) Recruitment of Tel1^ATM^ to telomeres in various mutant combinations among *nbs1* and *rad3*, monitored by quantitative ChIP assays. Mean values plus or minus one standard deviation for two to four independent experiments are plotted. Only *rad3-kdΔ*, *rad3Δ* and *rad3-kdΔ nbs1-c60Δ* showed higher mean % precipitated DNA (% ppt DNA) values compared to untagged control. Tel1 binding to telomeres in *rad3-kdΔ* and *rad3Δ* cells was deemed statistically insignificant (P values 0.155 and 0.139, respectively) compared to untagged control, due to large standard deviation in % ppt DNA values among four independent ChIP experiments. However, we consistently found that % ppt DNA values for *rad3-kdΔ* and *rad3Δ* cells are higher than untagged control or *rad3^+^* cells for a given set of ChIP experiment performed the same day. Tel1 binding to telomeres in *rad3-kdΔ nbs1-c60Δ* cells, compared to untagged control, was statistically significant (P = 0.016). Since myc-Tel1 expressed from its endogenous promoter could not be detected in whole cell extracts by anti-myc western blot, anti-myc immunoprecipitation (IP) was performed to enrich for myc-Tel1. Based on anti-myc western blot, comparable amounts of Tel1 were pulled down in different mutant backgrounds. Anti-Cdc2 western blots were used as loading controls.

To gain insights into how the Rad3-kdΔ^ATR^-Rad26^ATRIP^ complex contributes to telomere maintenance in *nbs1-c60Δ* cells, we next examined the association of the Rad3^ATR^-Rad26^ATRIP^ complex with fission yeast telomeres by quantitative chromatin immunoprecipitation (ChIP) assays. While a significant amount of telomeric DNA was found to associate with Rad26^ATRIP^ in *rad3^+^* and *rad3-kdΔ* cells, the amount of telomeric DNA precipitated by Rad26^ATRIP^ in *rad3Δ* cells was reduced to background levels (Figure 2A). Conversely, both wild-type Rad3^ATR^ and Rad3-kdΔ^ATR^ proteins associate with telomeric DNA in a Rad26-dependent manner, although the precipitation efficiency for Rad3-kdΔ^ATR^ was reduced compared to wild-type Rad3^ATR^ (Figure 2B). Taken together, these results are consistent with the notion that Rad26^ATRIP^ is essential for recruitment of Rad3^ATR^ to telomeres and that the N-terminal domain of Rad3^ATR^ contributes to the recruitment of Rad26^ATRIP^ to telomeres.

We also examined the recruitment of wild-type Nbs1 and Nbs1-c60Δ to telomeres in *rad3^+^*, *rad3-kdΔ*, and *rad3Δ* background by ChIP assays (Figure 2C). Since we were able to still detect a robust association of Nbs1-c60Δ with telomeres even in *rad3Δ* cells, we could exclude the possibility that the Rad3-kdΔ^ATR^-Rad26^ATRIP^ complex contributes to telomere maintenance by promoting recruitment of Nbs1-c60Δ to telomeres.

### The Rad3-kdΔ^ATR^-Rad26^ATRIP^ complex and the N-terminal domain of Nbs1 are both required for recruitment of Tel1^ATM^ to telomeres in the absence of Nbs1-Tel1^ATM^ interaction

Since expression of Rad3-kdΔ^ATR^ allowed cells to maintain stable telomeres in the absence of Nbs1-Tel1^ATM^ interaction but not in *tel1Δ* and *nbs1Δ* cells (Figure 1D), we hypothesized that Nbs1-c60Δ protein and the Rad3-kdΔ^ATR^-Rad26^ATRIP^ complex may function collaboratively to recruit Tel1^ATM^ to telomeres and maintain telomeres. Indeed, we detected robust Tel1^ATM^ recruitment to telomeres in *nbs1-c60Δ rad3-kdΔ* cells, but failed to detect Tel1^ATM^ at telomeres in *nbs1-c60Δ rad3Δ*, *nbs1Δ rad3-kdΔ*, and *nbs1Δ rad3Δ* cells (Figure 2D). The ability to recruit Tel1^ATM^ to telomeres in *rad3* mutant backgrounds (either *rad3-kdΔ* or *rad3Δ*) correlated perfectly with the telomere maintenance phenotypes of these mutant cells (Figure 1D and Figure 2D). Thus, our ChIP data establish that the N-terminal domain of Nbs1 and the telomere-bound Rad3-kdΔ^ATR^-Rad26^ATRIP^ complex must both be present, in order for Tel1^ATM^ to be recruited independently of the C-terminal Tel1^ATM^-interaction domain of Nbs1.

It should be noted that for those mutant strains that are defective in telomere maintenance and ultimately circularize their chromosomes, ChIP assays were performed with early generation cells prior to chromosome circularization by utilizing our recently described Rad3 plasmid loss system [18]. This system allows us to harvest cells immediately after the loss of Rad3^ATR^, when the average telomere length is comparable or slightly longer than in *rad3Δ* or *rad3-kdΔ* cells. Furthermore, by monitoring real-time PCR amplification cycle numbers for all ChIP input samples, we have ensured that mutant cells utilized in our experiments have not yet circularized their chromosomes, since the PCR primer-annealing sites would be completely lost upon chromosome circularization, resulting in a significant delay in PCR amplification.

In contrast to *nbs1-c60Δ* cells, *nbs1^+^* cells were able to recruit Tel1^ATM^ to telomeres in both *rad3-kdΔ* and *rad3Δ* backgrounds (Figure 2D). Thus, our ChIP data also support the notion that the C-terminal Tel1^ATM^ interaction domain of Nbs1 and the Rad3-kdΔ^ATR^-Rad26^ATRIP^ complex represent two pathways that are redundantly required for recruitment of Tel1^ATM^ to telomeres. Since we detect Tel1^ATM^ at telomeres only in cells carrying very short telomere-repeats (∼180 bp in *rad3-kdΔ* and *rad3Δ* cells), but not in cells with wild-type telomere-repeat length (∼280 bp in *rad3^+^* cells) (Figure 2D, Figure S1A), fission yeast Tel1^ATM^ may be preferentially recruited to short telomeres. Alternatively, active Rad3^ATR^ kinase might prevent association of Tel1^ATM^ with telomeres. In any case, our results establish that the C-terminal 60 amino acids of fission yeast Nbs1 are not essential for the recruitment of Tel1^ATM^ to critically short telomeres, due to a redundant non-kinase contribution of Rad3^ATR^-Rad26^ATRIP^ to Tel1^ATM^ recruitment.

### The N-terminal domain of Nbs1 contributes to the recruitment of Rad3^ATR^-Rad26^ATRIP^ to telomeres

**Figure 3:**
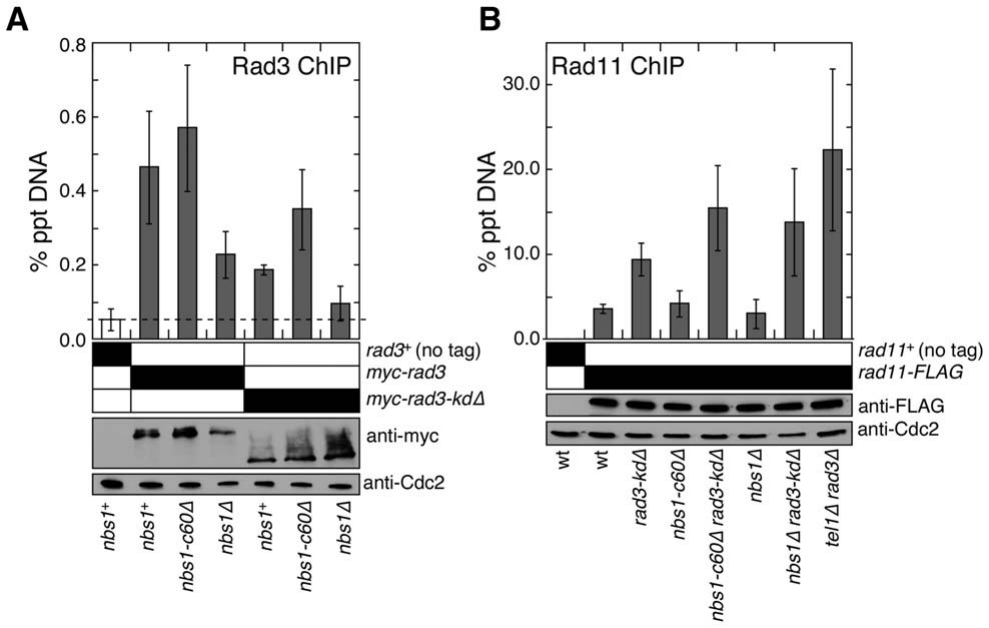
Nbs1 N-terminal domain is required to promote recruitment of the Rad3^ATR^-Rad26^ATRIP^ complex to telomeres. (A) Recruitment of wild-type Rad3^ATR^ or Rad3-kdΔ to telomeres in *nbs1^+^*, *nbs1-c60Δ* or *nbs1Δ* was monitored by quantitative ChIP assays. Averages and standard deviations from three to five independent ChIP experiments are plotted. Compared to untagged control, wild-type Rad3 was detected at telomeres in all genetic backgrounds tested (P values≤0.0003). Compared to *nbs1^+^* cells, wild-type Rad3 binding to telomeres was significantly reduced in *nbs1Δ* (P = 0.011) but not in *nbs1-c60Δ* (P = 0.316). Compared to untagged control, Rad3-kdΔ showed telomere recruitment in *nbs1^+^* and *nbs1-c60Δ* (P values≤0.0002) but not in *nbs1Δ* (P = 0.118). Based on anti-myc western blot, expression levels of wild-type or truncated Rad3^ATR^ are not affected by mutations in *nbs1*. Anti-Cdc2 western blots were used as loading controls. (B) Recruitment of RPA largest subunit Rad11 to telomeres, monitored by quantitative ChIP assays, did not change greatly among strains mutated for *nbs1* and *rad3*. Averages and standard deviations from three to six independent experiments are plotted. Compared to untagged control, Rad11 binding to telomeres was significant in all genetic backgrounds tested (P values≤0.01). Strains carrying *rad3-kdΔ* showed significantly higher telomere recruitment of Rad11 compared to *rad3^+^* cells for all *nbs1* alleles tested (P values≤0.01). No significant change in recruitment of Rad11 to telomeres among *rad3-kdΔ*, *nbs1-c60Δ rad3-kdΔ*, and *nbs1Δ rad3-kdΔ* cells was detected (P values≥0.06). Based on anti–FLAG western blot, expression levels of Rad11 are not affected by mutations in *nbs1* or *rad3*. Western blots with anti-Cdc2 were used as loading controls.

In order to better understand how the N-terminal domain of Nbs1 contributes to the Rad3-kdΔ^ATR^-dependent maintenance of telomeres in *nbs1-c60Δ* cells, we next examined the recruitment of Rad3^ATR^ or Rad3-kdΔ^ATR^ to telomeres in *nbs1^+^*, *nbs1-c60Δ* or *nbs1Δ* backgrounds by ChIP assays. As shown in Figure 3A, we found that *nbs1Δ* cells recruit significantly less wild-type Rad3 or Rad3-kdΔ^ATR^ proteins to telomeres compared to *nbs1^+^* or *nbs1-c60Δ* cells, suggesting that Nbs1-c60Δ protein contributes to the efficient recruitment of the Rad3^ATR^-Rad26^ATRIP^ complex to telomeres. Accordingly, the loss of the Rad3-kdΔ^ATR^-Rad26^ATRIP^ complex from telomeres in *nbs1Δ* cells might explain why *rad3-kdΔ nbs1Δ* cells fail to recruit Tel1^ATM^ to telomeres and circularize chromosomes (Figure 1D, Figure 2D, and Figure 3A). It should be noted that a substantial amount of wild-type Rad3^ATR^ could still be recruited to telomeres in *nbs1Δ* cells. In fact, since *nbs1Δ*, *rad50Δ*, *rad32Δ^mre11^* and *tel1Δ* cells all maintain essentially wild-type length or only slightly shorter telomeres in fission yeast [27],[33], it appears that the MRN complex and Tel1^ATM^ do not make significant contributions to telomere maintenance as long as cells express the wild-type Rad3^ATR^-Rad26^ATRIP^ complex.

The ATR-ATRIP complex preferentially binds to RPA-coated ssDNA, and the MRN complex promotes formation of 3′ ssDNA at DSBs and telomeres [10], [34]–[36]. Thus, we considered the possibility that the fission yeast MRN complex, independently of its Nbs1 C-terminal Tel1^ATM^ interaction domain, might promote recruitment of Rad3^ATR^-Rad26^ATRIP^ to telomeres by promoting accumulation of RPA-coated ssDNA at telomeres. However, we found comparable level of RPA (Rad11) associated with telomeric DNA in *rad3-kdΔ nbs1-c60Δ* and *rad3-kdΔ nbs1Δ* cells (Figure 3B). We also considered the possibility that the N-terminus of Nbs1 might contribute to the recruitment of Rad3^ATR^-Rad26^ATRIP^ to telomeres by promoting association between the MRN complex and the Rad3^ATR^-Rad26^ATRIP^ complex, since it was previously shown that mammalian MRN interacts with ATR-ATRIP [37]. However, we could not detect a direct interaction between Nbs1 and Rad3^ATR^ or between Nbs1 and Rad26^ATRIP^ by yeast 2-hybrid assays, and failed to detect an interaction between Nbs1 and Rad26^ATRIP^ by co-IP experiments (data not shown). Thus, further investigations are necessary to fully understand how the N-terminal domain of Nbs1 contributes to efficient recruitment of the Rad3^ATR^-Rad26^ATRIP^ complex to telomeres.

### Over-expression of Tel1^ATM^ can bypass the requirement for the Nbs1 C-terminal domain and Rad3^ATR^-Rad26^ATRIP^ complex in telomere maintenance

**Figure 4:**
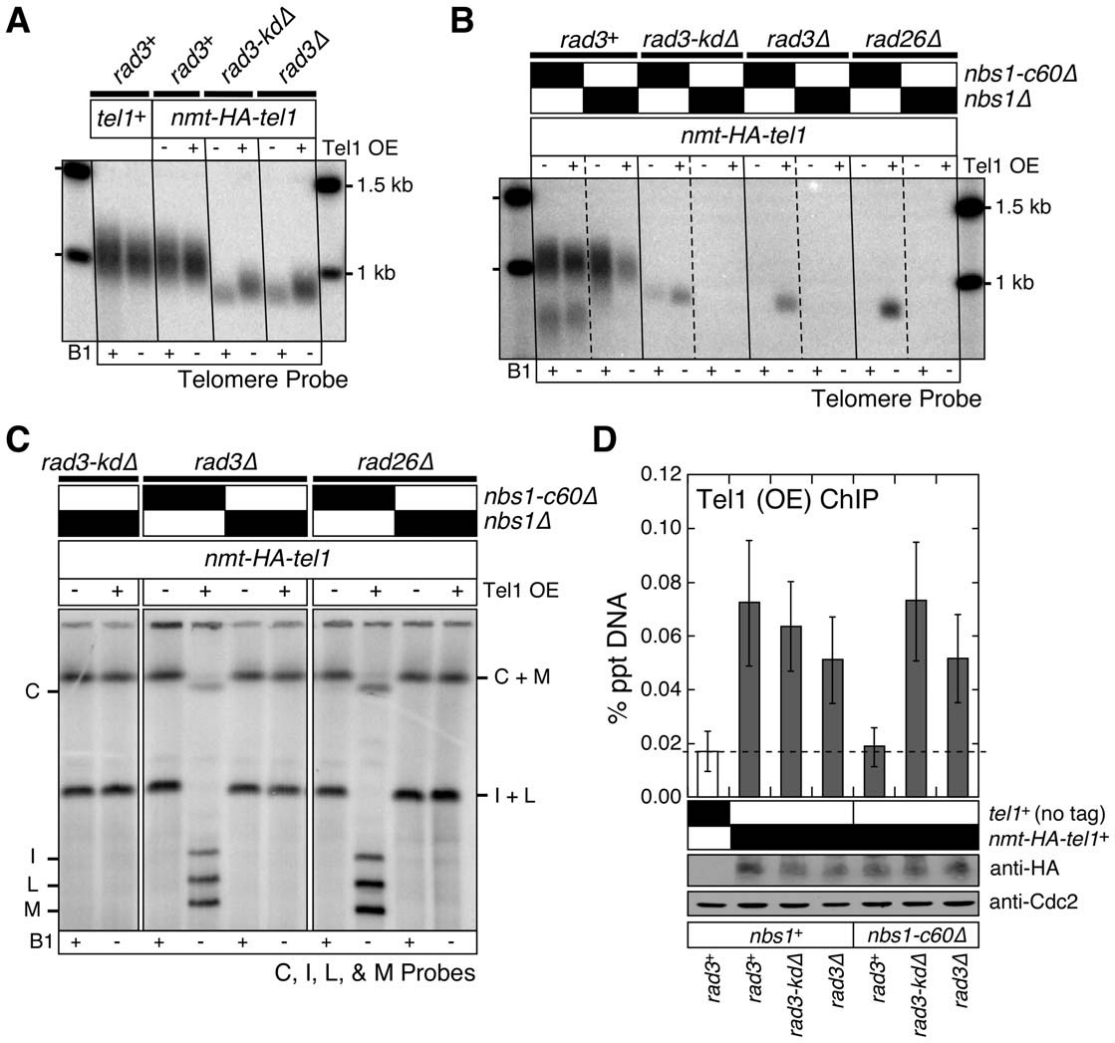
Over-expression of Tel1^ATM^ bypasses the Tel1^ATM^ recruitment function redundantly provided by the Nbs1 C-terminus and the Rad3^ATR^-Rad26^ATRIP^ complex, but does not bypass the complete deletion of Nbs1. (A, B) Southern blot analysis of EcoRI digested genomic DNA probed with a telomeric repeat specific probe for indicated strains. See Figure S1A for a schematic drawing of the fission yeast telomere restriction map. (C) Characterization of telomere status by PFGE for indicated strains. For all panels, fission yeast cells were grown in minimal media in the presence or absence of thiamine (B1) to repress (−) or induce (+) expression of Tel1 under the control of the *nmt1* ^+^ promoter respectively, for at least 150 cell divisions. (D) Recruitment of over-expressed Tel1^ATM^ to telomeres in various mutant combinations among *nbs1* and *rad3*, monitored by quantitative ChIP assays. Averages and standard deviations from three to four independent ChIP experiments are plotted. Compared to untagged control, over-expressed Tel1^ATM^ was detected at telomeres in all genetic backgrounds tested (P values ≤0.019), except in *rad3^+^ nbs1-c60Δ* (P = 0.81). Tel1^ATM^ was transiently over-expressed by growing cells in minimal media lacking thiamine for 21 hr prior to harvest. Based on anti-HA western blot, expression levels of over-expressed Tel1^ATM^ are comparable among all genetic backgrounds tested. Anti-Cdc2 western blots were used as loading controls.

Over-expression of Tel1^ATM^ results in MRX (Mre11-Rad50-Xrs2) complex-dependent elongation of telomeres in budding yeast [14],[38],[39], consistent with the notion that recruitment of Tel1^ATM^ to telomeres represents the critical rate limiting step in regulating telomere length in budding yeast. By contrast, over-expression of Tel1^ATM^ was unable to increase telomere length in wild-type fission yeast cells (Figure 4A), even though over-expressed Tel1^ATM^ can be detected at telomeres by ChIP assays (Figure 4D). However, we did observe a partial suppression of telomere shortening in *rad3-kdΔ* and *rad3Δ* cells upon Tel1^ATM^ over-expression (Figure 4A), suggesting that the level of Tel1^ATM^ recruitment to short telomeres becomes a critical limiting factor in telomere length determination in the absence of the functional Rad3^ATR^-Rad26^ATRIP^ complex. Interestingly, we also found that the Nbs1 C-terminal Tel1^ATM^ interaction domain is essential for over-expressed Tel1^ATM^ to associate with telomeres in *rad3^+^* cells, but dispensable in *rad3-kdΔ* and *rad3Δ* cells (Figure 4D). Thus, it appears that the presence of the kinase active Rad3^ATR^-Rad26^ATRIP^ complex can prevent recruitment of over-expressed Tel1^ATM^ to telomeres if cells are missing the C-terminal Tel1^ATM^ interaction domain of Nbs1.

We reasoned that over-expression of Tel1^ATM^ might be able to suppress chromosome circularization caused by simultaneous mutations in Nbs1 and Rad3^ATR^-Rad26^ATRIP^, if the essential telomere maintenance function, redundantly provided by the Nbs1 C-terminal Tel1^ATM^ interaction domain and the kinase-inactive Rad3^ATR^-Rad26^ATRIP^ complex, were to promote efficient recruitment of Tel1^ATM^ to telomeres. Indeed, over-expression of Tel1^ATM^ allowed recruitment of Tel1^ATM^ in *nbs1-c60Δ rad3Δ* cells (Figure 4D) and suppressed chromosome circularization in *nbs1-c60Δ rad3Δ* and *nbs1-c60Δ rad26Δ* cells (Figure 4B and 4C). However, over-expression of Tel1^ATM^ was unable to suppress chromosome circularization in *nbs1Δ rad3-kdΔ*, *nbs1Δ rad3Δ* and *nbs1Δ rad26Δ* cells, indicating that the expression of Nbs1 (and likely other subunits of the MRN complex) is essential for telomere maintenance in the absence of kinase-active Rad3^ATR^-Rad26^ATRIP^ even when Tel1^ATM^ is over-expressed. Taken together, our results indicate that the Nbs1 C-terminal Tel1^ATM^ interaction domain and the kinase-independent function of the Rad3-kdΔ^ATR^-Rad26^ATRIP^ complex redundantly contribute to efficient recruitment of Tel1^ATM^ to telomeres, when Tel1^ATM^ is expressed at endogenous level. Our results further demonstrate that when Tel1^ATM^ is over-expressed, the MRN complex can contribute to telomere maintenance even in the absence of both the C-terminal Tel1^ATM^ interaction domain of Nbs1 and the Rad3^ATR^-Rad26^ATRIP^ complex.

### Over-expression of Rad3^ATR^ can bypass the requirement for Rad26^ATRIP^ and Nbs1, but not Tel1^ATM^ in telomere maintenance

**Figure 5:**
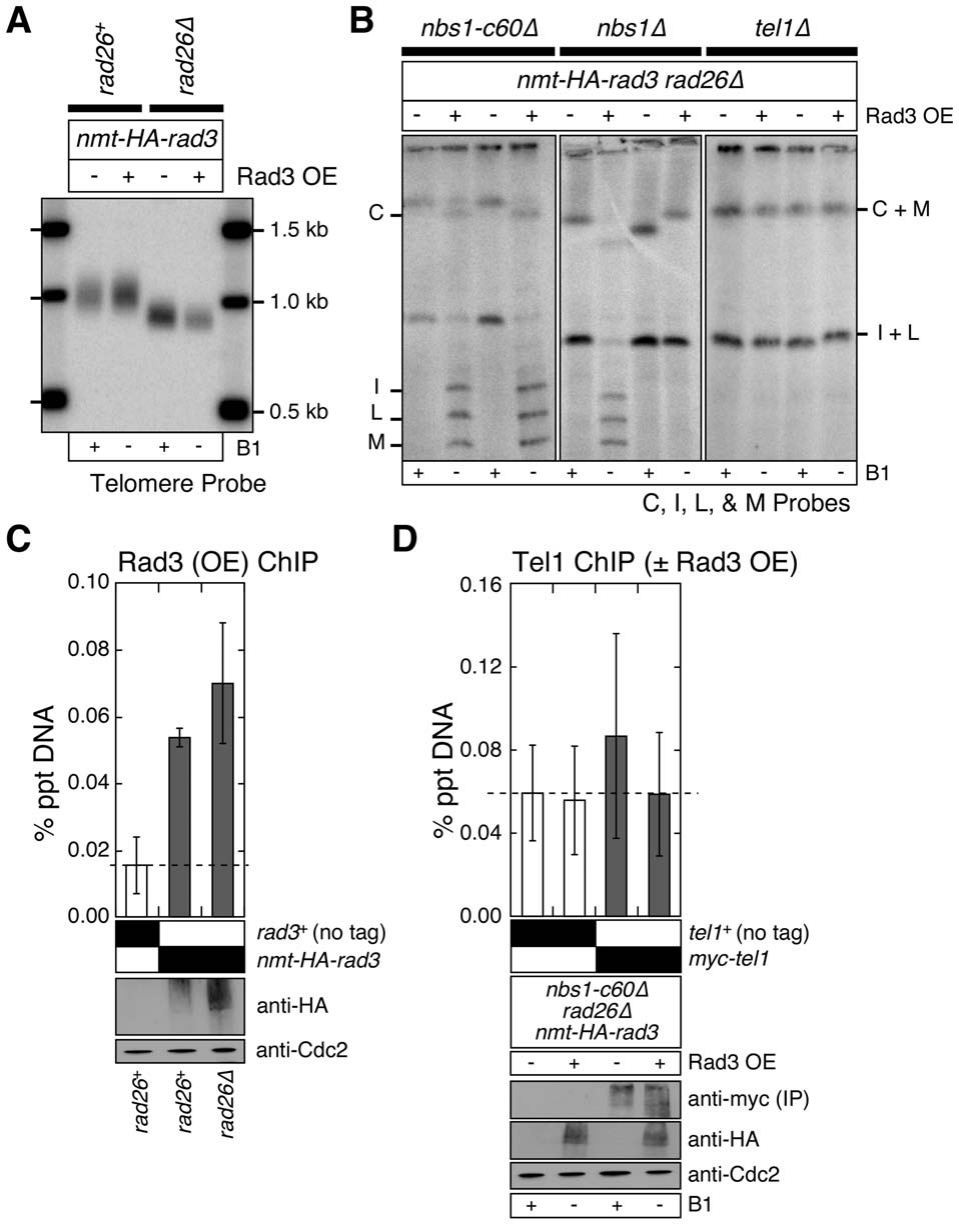
Over-expression of Rad3^ATR^ can bypass the Rad3^ATR^ recruitment function provided by Rad26^ATRIP^ and Nbs1. (A) Southern blot analysis of EcoRI digested genomic DNA, hybridized to telomere repeat specific probe. Fission yeast cells were grown in minimal media in the presence or absence of thiamine (B1) to repress (−) or induce (+) expression of Rad3 under the control of the *nmt1* ^+^ promoter, respectively, for at least 150 cell divisions. See Figure S1A for a schematic drawing of the fission yeast telomere restriction map. (B) Characterization of telomere status by PFGE for indicated strains. Fission yeast cells were grown as in (A). Two independent clones of each genotype are shown. Rad3 over-expression in *nbs1Δ rad26Δ* cells resulted in mixed telomere phenotypes, suggesting partial suppression of telomere dysfunction. (C) Recruitment of over-expressed Rad3^ATR^ to telomeres in *rad26^+^* or *rad26Δ* cells, monitored by quantitative ChIP assays. Averages and standard deviations from three independent ChIP experiments are plotted. Compared to untagged control, over-expressed Rad3^ATR^ was detected at telomeres in both *rad26^+^* (P = 0.0016) and *rad26Δ* (P = 0.0087) cells. Rad3^ATR^ was transiently over-expressed by growing cells in minimal media lacking thiamine for 21 hr prior to harvest. Expression levels of over-expressed Rad3^ATR^ are comparable, based on anti-HA western blot. Anti-Cdc2 western blots were used as loading controls. (D) Recruitment of endogenous Tel1^ATM^ to telomeres in *nbs1-c60Δ rad26Δ* cells, with or without over-expressed Rad3^ATR^, was monitored by quantitative ChIP assays. Averages and standard deviations from three independent ChIP experiments are plotted. No significant binding of Tel1^ATM^ to telomeres above untagged control was observed. Rad3^ATR^ was transiently over-expressed as in (C). Anti-HA western blots were used to monitor expression levels of over-expressed Rad3^ATR^. Since myc-Tel1 expressed from its endogenous promoter could not be detected in whole cell extracts by anti-myc western blot, anti-myc immunoprecipitation (IP) was performed to enrich for myc-Tel1. Anti-Cdc2 western blots were used as loading controls.

Our ChIP data indicated that both the Rad26^ATRIP^ regulatory subunit and the N-terminal domain of Nbs1 play critical roles in promoting recruitment of Rad3^ATR^ to telomeres (Figure 2B, Figure 3A). Therefore, we next investigated if over-expression of Rad3^ATR^ might be able to bypass the requirement for Rad26^ATR^ and/or Nbs1 in telomere maintenance. Based on ChIP analysis, over-expression of Rad3^ATR^ bypassed the requirement for Rad26^ATRIP^ in recruitment of Rad3^ATR^ to telomeres (Figure 5C). However, over-expression of Rad3^ATR^ was not able to suppress telomere shortening observed in *rad26Δ* cells (Figure 5A). Thus, in the presence of wild-type Nbs1 (and thus intact Nbs1-Tel1^ATM^ interaction), forced recruitment of Rad3^ATR^ to telomeres by over-expression is not sufficient to elongate telomeres in *rad26Δ* cells. On the other hand, we found that Rad3^ATR^ over-expression completely suppressed chromosome circularization in *rad26Δ nbs1-c60Δ* cells (Figure 5B), consistent with the notion that the essential telomere maintenance function contributed by Rad26^ATRIP^ in *nbs1-c60Δ* is to promote recruitment of Rad3^ATR^ to telomeres.

Even when Rad3^ATR^ is over-expressed, the N-terminus of Nbs1 still appears to contribute to telomere maintenance in *rad26Δ* cells, since suppression of chromosome circularization by over-expression of Rad3^ATR^ was much more complete in *rad26Δ nbs1-c60Δ* cells than in *rad26Δ nbs1Δ* cells (Figure 5B). Since we observed a mixed telomere phenotype among survivor cells after extensive restreaking of *nbs1Δ rad26Δ* cells over-expressing Rad3^ATR^ on agar plates, we concluded that over-expression of Rad3^ATR^ can partially bypass the essential telomere function of Nbs1 in the absence of Rad26^ATRIP^ (Figure 5B). The observed partial suppression of chromosome circularization in *nbs1Δ rad26Δ* by over-expression of Rad3^ATR^ is consistent with the notion that the N-terminal domain of Nbs1 contributes to telomere maintenance by promoting recruitment of Rad3^ATR^ to telomeres.

By contrast, Rad3^ATR^ over-expression was unable to suppress chromosome circularization observed in *rad26Δ tel1Δ* cells (Figure 5B). Thus, it appears that over-expression of Rad3^ATR^ allows the Tel1^ATM^-dependent mechanism to maintain telomeres in *rad26Δ nbs1-c60Δ* or *rad26Δ nbs1Δ* cells. One possible mechanism by which Rad3^ATR^ over-expression might promote Tel1^ATM^-dependent telomere maintenance in *rad26Δ nbs1-c60Δ* or *rad26Δ nbs1Δ* cells is to promote recruitment of Tel1^ATM^ to telomeres. Therefore, we examined changes in Tel1^ATM^ recruitment to telomeres in *nbs1-c60Δ rad26Δ* cells with or without over-expression of Rad3^ATR^ by ChIP analyses. However, we were unable to detect Rad3^ATR^ over-expression dependent recruitment of Tel1^ATM^ to telomeres (Figure 5D). Thus, further investigations are necessary to fully understand how over-expression of Rad3^ATR^ contributes to the Tel1^ATM^-dependent suppression of chromosome circularization in fission yeast cells simultaneously lacking functional Rad26^ATRIP^ and Nbs1.

## Discussion

**Figure 6:**
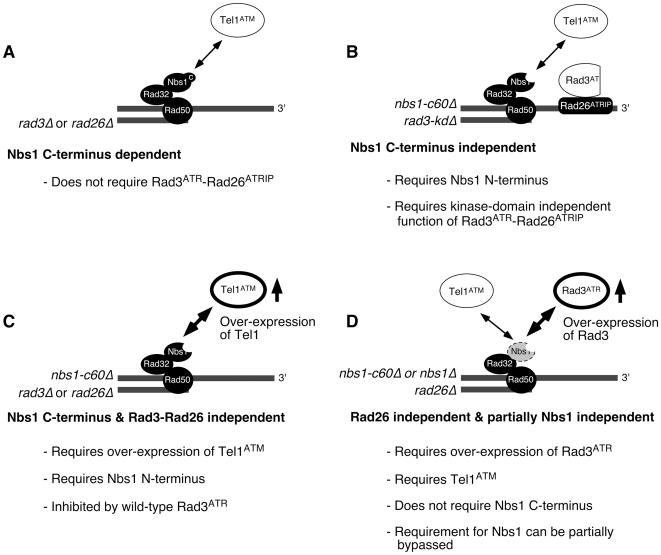
Summary of mechanisms controlling the recruitment of Rad3^ATR^-Rad26^ATRIP^ and Tel1^ATM^ to telomeres in fission yeast. See main text for details. Over-expressed Tel1^ATM^ (C) and Rad3^ATR^ (D) are emphasized with thicker lines for their respective ovals.

In the current study, we investigated how two PIKK-containing complexes, Tel1^ATM^-MRN and Rad3^ATR^-Rad26^ATRIP^, contribute to telomere length regulation in fission yeast (summarized in Figure 6). We have demonstrated that fission yeast Tel1^ATM^ can be recruited to telomeres by two alternative and redundant mechanisms, which are either dependent or independent of the Nbs1 C-terminal Tel1^ATM^ interaction domain (Figure 6A and 6B). Our analyses indicated that the Nbs1 C-terminus dependent mode of Tel1^ATM^ recruitment to telomeres does not require the Rad3^ATR^-Rad26^ATRIP^ complex, and in fact, it might be inhibited by the presence of wild-type Rad3^ATR^-Rad26^ATRIP^ (Figure 2D). On the other hand, the Nbs1 C-terminus independent mode of Tel1^ATM^ recruitment requires the presence of telomere bound kinase-inactive Rad3-kdΔ^ATR^-Rad26^ATRIP^ complex (Figure 2A, 2B, and 2D). Since the N-terminal domain of Nbs1 is essential for recruitment of the Rad3-kdΔ^ATR^-Rad26^ATRIP^ complex to telomeres (Figure 3A), all our results are consistent with the notion that association of Rad3-kdΔ^ATR^-Rad26^ATRIP^ to telomeres is the crucial determinant that allows recruitment of Tel1^ATM^ to telomeres in the absence of the Nbs1 C-terminal Tel1^ATM^ interaction domain. The notion that the C-terminus of Nbs1 and the Rad3-kdΔ^ATR^-Rad26^ATRIP^ complex redundantly contribute to the recruitment of Tel1^ATM^ is also supported by our finding that over-expression of Tel1^ATM^ can entirely bypass the requirement for Rad3^ATR^-Rad26^ATRIP^ for telomere maintenance and Tel1^ATM^ recruitment to telomeres in *nbs1-c60Δ* cells (Figure 4, Figure 6C). Moreover, the finding that over-expression of Rad3^ATR^ was able to at least partially suppress the loss of Nbs1 protein (Figure 5B, Figure 6D) provided further support for the notion that the N-terminal domain of Nbs1 also contributes to the recruitment of Rad3^ATR^ to telomeres.

Thus, our findings are generally consistent with the model that at least one PIKK activity needs to be localized at telomeres, in order to stably maintain telomeres in fission yeast, although we have not yet tested if Tel1^ATM^ kinase activity is indeed required for telomere maintenance in *nbs1-c60Δ rad3-kdΔ* cells. It is currently unknown which telomere-associated protein(s) represent critical substrate(s) of Tel1/Rad3 that are essential for telomere maintenance. However, we were able to demonstrate recently that Tel1^ATM^ and Rad3^ATR^ are redundantly required to prevent accumulation of homologous recombination DNA repair factors and RPA at telomeres, to promote efficient telomere recruitment of Tpz1 and Ccq1 (subunits of the Pot1 telomere capping complex), and to recruit telomerase to telomeres [18].

Our results also indicate that fission yeast Tel1^ATM^ is recruited preferentially to short telomeres (Figure 2D). Due to the kinase-independent function of the Rad3^ATR^-Rad26^ATRIP^ complex, the Nbs1 C-terminal 60 amino acid Tel1^ATM^ interaction domain is dispensable for recruitment of Tel1^ATM^ to short telomeres. Tel1^ATM^ is also recruited preferentially to short telomeres in budding yeast [14],[38],[40]. Based on studies utilizing *xrs2-664* mutant cells, which express the truncated Xrs2^Nbs1^ protein lacking the C-terminal 190 amino acids (full length 884 amino acids), it was suggested that the Xrs2^Nbs1^ C-terminal Tel1^ATM^ interaction domain is essential for recruitment of Tel1^ATM^ to critically short telomeres [14]. However, a recent study has shown that, while deleting as little as 20 amino acids from C-terminus of Xrs2^Nbs1^ is sufficient to disrupt the interaction between Tel1^ATM^ and Xrs2^Nbs1^, *xrs2* mutant cells lacking the C-terminal 20 amino acids maintain significantly longer telomeres than *xrs2-664* or *xrs2Δ* cells [41]. Therefore, budding yeast Xrs2^Nbs1^ also appears to contribute to telomere maintenance independently of its C-terminal Tel1^ATM^ interaction domain. Thus, it would be interesting to test if the N-terminal domain of Xrs2^Nbs1^ might also collaborate with the Mec1^ATR^-Ddc2^ATRIP^ complex, perhaps independently of Mec1^ATR^ kinase activity, in regulating Tel1^ATM^ recruitment to telomeres in budding yeast.

Our ChIP data indicate that very little or no Tel1^ATM^ is recruited to telomeres in wild-type fission yeast cells. On the other hand, Rad3^ATR^-Rad26^ATRIP^ is specifically recruited to replicating telomeres and appears to act as the primary sensor of transiently “open” telomeres during S-phase [15]. Therefore, it makes sense that *rad3Δ* or *rad26Δ* cause a more severe shortening of telomeres than *tel1Δ* in fission yeast [25],[27],[42]. Thus, Tel1^ATM^ is likely to function as a back-up mechanism to extend critically short telomeres in fission yeast. In contrast, budding yeast *mec1Δ* cells show very little shortening of telomeres [43], while *tel1Δ* or *xrs2Δ* cells carry extremely short telomeres [44],[45]. It has also been reported that budding yeast Mec1^ATR^ cannot be detected at telomeres by ChIP in wild-type cells [13]. However, simultaneous loss of Mec1^ATR^-Ddc2^ATRIP^ and Tel1^ATM^-MRX pathways results in a severe defect in telomere maintenance [43],[46], much like in fission yeast cells. Thus, available data suggest that budding yeast telomeres are primarily regulated by Tel1^ATM^-MRX, and Mec1^ATR^-Ddc2^ATRIP^ fulfills a back-up role for maintaining telomeres.

There are intriguing similarities between our current findings and findings in mammalian cells, where ATR-ATRIP was found to act upstream of ATM. In response to replication stress or UV irradiation during S-phase, the Nbs1 C-terminal ATM interaction domain is dispensable, but the N-terminus of Nbs1 is essential, for ATR to activate ATM in mammalian cells [12]. However, it is currently unknown whether ATR-ATRIP might also contribute to the recruitment of ATM to sites of stalled or distressed DNA replication forks in a kinase-independent manner, besides the previously established kinase-dependent role of ATR in converting inactive ATM dimers into active monomers [12]. However, the similar requirement for the N-terminus, but not the C-terminus, of Nbs1 for the ATR-ATM crosstalk in response to DNA replication stress in mammalian cells and telomere maintenance in fission yeast might suggest that fission yeast telomeres may be recognized primarily as stressed and/or abnormal replication forks by ATM/ATR kinases.

Studies in mammalian cells have also established the existence of an ATM-ATR crosstalk in response to DNA DSBs, where ATM and the MRN complex contribute to the recruitment of ATR-ATRIP to DSBs by promoting the generation of RPA-coated ssDNA at DSB sites [9]–[11],[47],[48]. The Nbs1 C-terminal ATM interaction domain is required to recruit ATM to DSBs induced by ionizing radiation (IR) and to promote ATM-dependent phosphorylation events [5],[6],[49], and it is thus critical for the ATM-ATR crosstalk in response to DSBs. While the critical role of Tel1^ATM^ in preferentially extending short telomeres in budding yeast has been well established [14],[38], mouse ATM was found not to be critical for extending short telomeres [50]. This difference might indicate that mouse ATR-ATRIP and ATM-MRN pathways work redundantly in extending critically short telomeres. Alternatively, since recruitment of human ATR to telomeres was found to occur earlier in S-phase than recruitment of ATM to telomeres [16], mammalian ATR-ATRIP may function upstream of ATM in telomere length maintenance, and perhaps also possess kinase-independent functions in recruitment of ATM to telomeres. While further analyses are clearly needed to test these speculations, our current findings highlight a complex molecular crosstalk between ATM-MRN and ATR-ATRIP pathways in recognizing an “open” configuration of telomeres to allow their stable maintenance.

## Materials and Methods

### Reagents and general methods

Fission yeast strains used in this study were constructed by standard techniques [51] and are listed in Table S1. Primers listed in Table S2 were used to construct new strains. For *nbs1-myc*, *nmt-HA-rad3*, *myc-rad26* and *YFP-rad26*, original strains were described previously [15],[24],[27],[52]. For *myc-rad3*, *nbs1-c60Δ-myc*, *myc-tel1* and *nmt-HA-tel1*, PCR-based methods [53],[54] were used to generate tagged strains. For *nbs1Δ::kanMX*, *nbs1-c60Δ::kanMX6*, *rad3-kdΔ::ura4^+^*, *rad3Δ::LEU2* and *rad26Δ::ura4^+^*, original strains were described previously [5], [25]–[27],[55]. For *nbs1Δ::natMX*, PCR-based methods [53],[54] were used to generate deletion strains. Budding yeast strains used in yeast two-hybrid assays are also listed in Table S1. Plasmids used in this study are listed in Table S3. Yeast two hybrid assays were performed by mating *S. cerevisiae MAT*a strains harboring GAL4-DBD plasmids with *MAT*α strains harboring GAL4-AD plasmids, as described in the MATCHMAKER system manual (Clonetech). Positive two-hybrid interactions were identified by spotting mated cells onto SD-HTL plates. Sensitivities of fission yeast cells to IR, UV, HU (hydroxyurea) and CPT (camptothecin) were assayed as previously described [24].

### Co-immunoprecipitation and western blot analyses

For most strains, cell extracts were prepared in lysis buffer 1 [50mM Tris pH8.0, 150mM NaCl, 10% glycerol, 5mM EDTA, 0.5% NP40, 50mM NaF, 1mM DTT, 1mM PMSF, 1mM Na_3_VO_4_, ‘Complete’ protease inhibitor cocktail (Roche)], either by glass bead disruption using FastPrep homogenizer (MP Biomedical) or by cryogenic disruption using MM301 Ball Mill (Retsch). For strains expressing myc-Rad3, myc-Rad3-kdΔ or myc-Tel1, lysis buffer 2 [25mM Tris pH7.5, 100mM NaCl, 10% glycerol, 15mM EDTA, 0.1% NP40, 1% Triton, 15mM MgCl_2_, 0.1mM NaF, 0.5mM DTT, 1mM PMSF, 1mM Na_3_VO_4_, ‘Complete’ protease inhibitor cocktail] was used. For co-immunoprecipitation analyses, proteins were immunoprecipitated using either monoclonal anti-myc antibody (9B11, Cell Signaling) or monoclonal anti-HA antibody (12CA5, Roche), and protein G Dynabeads (Invitrogen) or protein G sepharose beads (GE) respectively. Proteins in whole cell extract or from immunoprecipitations were analyzed by western blots using monoclonal anti-HA antibody (12CA5), monoclonal anti-myc antibody (9B11), monoclonal anti-FLAG antibody (M2, F1804, Sigma) or monoclonal anti-GFP antibody (7.1/B.1, Roche). Anti-Cdc2 antibody (y100.4, Abcam) was used for loading control.

### Pulsed-field gel electrophoresis (PFGE)

Chromosomal DNA samples were prepared in agarose plugs, digested with NotI restriction enzyme, and fractionated in 1% agarose gels using the CHEF-DR III system (Bio-Rad) as previously described [25]. C, I, L, and M probes specific for telomeric NotI fragments were prepared as previously described [56]. Except for Tel1 or Rad3 over-expression experiments, cells were extensively restreaked on YES agar plates to achieve terminal telomere states prior to harvesting. For over-expression experiments, minimal media was used for *nmt1^+^* promoter-controlled over-expression.

### Southern blot analyses

*S. pombe* genomic DNA samples were digested with EcoRI or ApaI, separated on 1.2% (EcoRI) or 2% (ApaI) agarose, transferred to Hybond-XL membrane (GE), and hybridized to telomere probe [57] in Church Buffer [0.25M sodium phosphate buffer pH7.2, 1mM EDTA, 1% BSA, 7% SDS] at 65°C overnight to monitor telomere length.

### ChIP assays

Cells were processed for ChIP and analyzed as previously described [15], using either monoclonal anti-myc (9B11; Cell Signaling), anti-FLAG (M2, F1804, Sigma), or anti-HA (12CA5) antibodies. Percent precipitated DNA values (% ppt DNA) were calculated based on ΔCt between Input and IP samples after performing several independent triplicate SYBR Green-based real-time PCR (Bio-Rad) using telomere primers jk380 and jk381 [15]. For genetic backgrounds that cause eventual circularization of chromosomes due to a telomere maintenance defect, a Rad3 plasmid (pREP41H-rad3) was utilized to maintain linear chromosomes during strain construction [18]. Prior to ChIP experiments, single colonies that had lost the Rad3 plasmid were selected based on lack of growth on media lacking histidine and sensitivity to HU, and immediately utilized in ChIP experiments. Based on Southern blot analysis, the early generation strains that have just lost the Rad3 plasmid carry comparable or slightly longer telomeres than *rad3Δ* cells [18] (data not shown).

### Statistical analysis

In order to determine statistical significance of our data, two-tailed Student's t-tests were performed, and P values ≤0.05 were considered as statistically significant differences.

## Supporting Information

Figure S1 Similar telomere length and DNA damage sensitivities for *rad3-kd*Δ and *rad3*Δ cells. (A) Southern blot analysis of ApaI digested genomic DNA, hybridized to telomere repeat specific probe. A fission yeast telomere restriction map for telomeric and sub-telomeric regions cloned in pNSU70 plasmid [Sugawara NF (1988) DNA sequences at the telomeres of the fission yeast *S. pombe*. (Ph.D. Thesis). Cambridge, Massachusetts: Harvard University.] is shown on the right. (B) Five-fold serial dilutions of wild-type and various mutant strains for *rad3* and *nbs1* plated onto YES media with indicated concentrations of HU or CPT. Pictures were taken after 3 days at 32°C. (C–F) Survival of wild-type and various mutant cells after exposure to indicated doses of IR or UV. Surviving colonies on YES plates were counted after 3 days at 32°C.
(1.43 MB TIF)

Table S1 Yeast strains used in this study.
(0.20 MB DOC)

Table S2 DNA primers used in strain construction.
(0.06 MB DOC)

Table S3 Plasmids used in this study.
(0.04 MB DOC)
