# Supplementary figures and images for: A Kinase-Independent Role for the Rad3ATR-Rad26ATRIP Complex in Recruitment of Tel1ATM to Telomeres in Fission Yeast
Source: PLoS Genet. 2010 Feb 5;6(2):e1000839. doi: 10.1371/journal.pgen.1000839 (PMC2816689; doi:10.1371/journal.pgen.1000839)

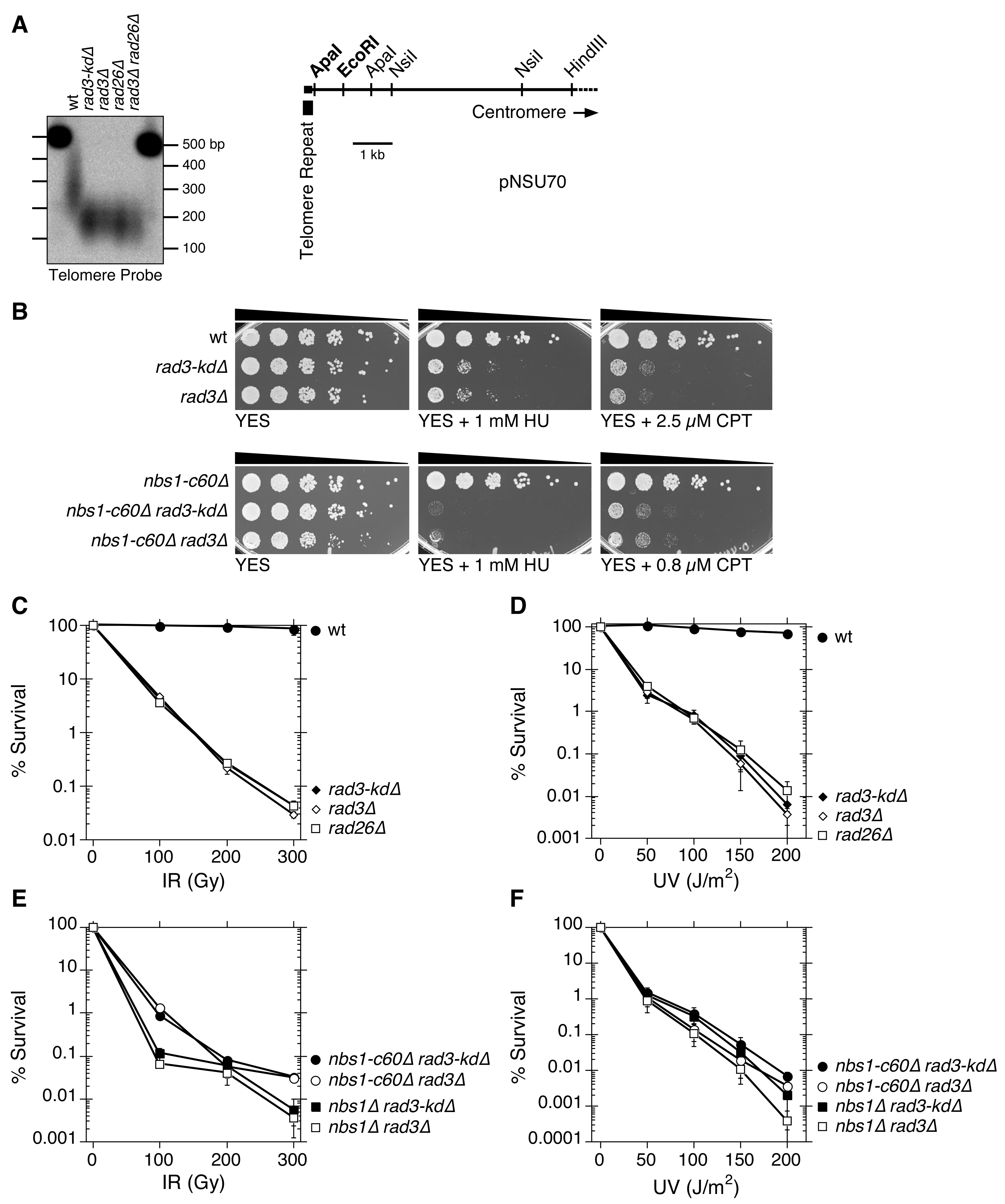

Supplement: Figure S1 — Similar telomere length and DNA damage sensitivities for rad3-kdΔ and rad3Δ cells. (A) Southern blot analysis of ApaI digested genomic DNA, hybridized to telomere repeat specific probe. A fission yeast telomere restriction map for telomeric and sub-telomeric regions cloned in pNSU70 plasmid [Sugawara NF (1988) DNA sequences at the telomeres of the fission yeast S. pombe. (Ph.D. Thesis). Cambridge, Massachusetts: Harvard University.] is shown on the right. (B) Five-fold serial dilutions of wild-type and various mutant strains for rad3 and nbs1 plated onto YES media with indicated concentrations of HU or CPT. Pictures were taken after 3 days at 32°C. (C–F) Survival of wild-type and various mutant cells after exposure to indicated doses of IR or UV. Surviving colonies on YES plates were counted after 3 days at 32°C. (1.43 MB TIF) [file pgen.1000839.s001.tif]
