# Supplementary material for: A Kinase-Independent Role for the Rad3ATR-Rad26ATRIP Complex in Recruitment of Tel1ATM to Telomeres in Fission Yeast
Source: PLoS Genet. 2010 Feb 5;6(2):e1000839. doi: 10.1371/journal.pgen.1000839 (PMC2816689; doi:10.1371/journal.pgen.1000839)
Supplement: Table S1 — Yeast strains used in this study. (0.20 MB DOC) [file pgen.1000839.s002.doc]

| **Table S1.** Yeast strains used in this study. | | |
| --- | --- | --- |
| **Figure** | **Strain** | **Genotype** |
| 1D | LS7837 | *h^+^ leu1-32 ura4-D18 his3-D1 nbs1^+^::GFP-ura4^+^* |
|  | LS4569 | *h^-^ leu1-32 ura4-D18 his3-D1 nbs1-c60∆::GFP-kanMX6* |
|  | TN2389 | *h^-^ leu1-32 ura4-D18 ade6-M216 his3-D1 nbs1∆::kanMX6* |
|  | LS8743 | *h^-^ leu1-32 ura4-D18 his3-D1 rad3-kd∆::ura4^+^ nbs1^+^::GFP-ura4^+^* |
|  | LS4570 | *h^-^ leu1-32 ura4-D18 his3-D1 rad3-kd∆::ura4^+^ nbs1-c60∆::GFP-kanMX6* |
|  | LS4572 | *h^-^ leu1-32 ura4-D18 his3-D1 rad3-kd∆::ura4^+^ nbs1-c60∆::GFP-kanMX6* |
|  | TN2915 | *h^+^ leu1-32 ura4-D18 rad3-kd∆::ura4^+^ nbs1∆::kanMX6* |
|  | LS7753 | *h^+^ leu1-32 ura4-D18 ade6-M210 his3-D1 rad3∆::LEU2 nbs1^+^::GFP-ura4^+^* |
|  | LS8736 | *h^-^ leu1-32 ura4-D18 ade6-M216 his3-D1 rad3∆::LEU2 nbs1-c60∆::GFP-kanMX6* |
|  | LS8410 | *h^-^ leu1-32 ura4-D18 his3-D1 rad3∆::LEU2 nbs1-c60∆::GFP-kanMX6* |
|  | LS8816 | *h^+^ leu1-32 ura4-D18 ade6-M216 his3-D1 rad3∆::LEU2 nbs1∆::kanMX6* |
|  | LS7745 | *h^-^ leu1-32 ura4-D18 ade6-M216 his3-D1 rad26∆::ura4^+^ nbs1^+^::GFP-ura4^+^* |
|  | LS5031 | *h^-^ leu1-32 ura4-D18 his3-D1 rad26∆::ura4^+^ nbs1-c60∆::GFP-kanMX6* |
|  | LS5032 | *h^-^ leu1-32 ura4-D18 his3-D1 rad26∆::ura4^+^ nbs1-c60∆::GFP-kanMX6* |
|  | LS8817 | *h^+^ leu1-32 ura4-D18 ade6-M216 his3-D1 rad26∆::ura4^+^ nbs1∆::kanMX6* |
|  |  |  |
| 1E | TN2411 | *h^-^ leu1-32 ura4-D18 his3-D1* |
|  | TN2961 | *h^+^ leu1-32 ura4-D18 his3-D1 rad26∆::ura4^+^::2YFP-rad26^+^-leu1^+^* |
|  | LS8777 | *h^-^ leu1-32 ura4-D18 his3-D1 9myc::rad3^+^ rad26∆::ura4^+^::2YFP-rad26^+^-leu1^+^* |
|  | LS8774 | *h^-^ leu1-32 ura4-D18 his3-D1 9myc::rad3-kd∆::kanMX4 rad26∆::ura4^+^::2YFP-rad26^+^-leu1^+^* |
|  |  |  |
| 1F | LS6597 | *MAT*α *gal4∆ gal80∆ his3 trp1-901 ade2-101 ura3-52 leu2-3 leu2-112 URA3::GAL1🡪lacZ met- // pGAD424-rad26 (LEU2; GAL4-AD:rad26^+^)* |
|  | LS6593 | *MAT*a *trp1-901 leu2-3 leu2-112 ura3-52 his3-200 gal4∆ gal80∆ LYS2::GAL1(UAS)-GAL1(TATA)-HIS3 GAL2(UAS)-GAL2(TATA)-ADE2 URA3::MEL1(UAS)-MEL1(TATA)-lacZ // pAS1-rad3 (TRP1; GAL4-DNA-BD:rad3^+^)* |
|  | LS8642 | *MAT*a *trp1-901 leu2-3 leu2-112 ura3-52 his3-200 gal4∆ gal80∆ LYS2::GAL1(UAS)-GAL1(TATA)-HIS3 GAL2(UAS)-GAL2(TATA)-ADE2 URA3::MEL1(UAS)-MEL1(TATA)-lacZ // pAS1-rad3-kd∆ (TRP1; GAL4-DNA-BD:rad3-kd∆)* |
|  | LS8644 | *MAT*a *trp1-901 leu2-3 leu2-112 ura3-52 his3-200 gal4∆ gal80∆ LYS2::GAL1(UAS)-GAL1(TATA)-HIS3 GAL2(UAS)-GAL2(TATA)-ADE2 URA3::MEL1(UAS)-MEL1(TATA)-lacZ // pAS1-rad3(1-191) (TRP1; GAL4-DNA-BD:rad3(1-191))* |
|  |  |  |
| 2A | TN2411 | *h^-^ leu1-32 ura4-D18 his3-D1* |
|  | LS7680 | *h^-^ leu1-32 ura4-D18 his3-D1 9myc::rad26^+^::hphMX6* |
|  | TN7845 | *h^-^ leu1-32 ura4-D18 his3-D1 rad3-kd∆::ura4^+^ 9myc::rad26^+^::hphMX6* |
|  | TN7849 | *h^-^ leu1-32 ura4-D18 his3-D1 rad3∆::LEU2 9myc::rad26^+^::hphMX6* |
|  |  |  |
| 2B | TN2411 | *h^-^ leu1-32 ura4-D18 his3-D1* |
|  | LS7938 | *h^-^ leu1-32 ura4-D18 his3-D1 9myc::rad3^+^* |
|  | LS8266 | *h^-^ leu1-32 ura4-D18 his3-D1 9myc::rad3^+^ rad26∆::ura4^+^* |
|  | LS8640 | *h^-^ leu1-32 ura4-D18 his3-D1 9myc::rad3-kd∆::kanMX4* |
|  | LS8771 | *h^-^ leu1-32 ura4-D18 his3-D1 9myc::rad3-kd∆::kanMX4 rad26∆::ura4^+^* |
|  |  |  |
| 2C | TN2411 | *h^-^ leu1-32 ura4-D18 his3-D1* |
|  | TN4362 | *h^-^ leu1-32 ura4-D18 his3-D1 nbs1^+^::13myc-kanMX6* |
|  | TN4367 | *h^-^ leu1-32 ura4-D18 his3-D1 rad3-kd∆::ura4^+^ nbs1^+^::13myc-kanMX6* |
|  | TN7758 | *h^-^ leu1-32 ura4-D18 his3-D1 rad3∆::LEU2 nbs1^+^::13myc-kanMX6* |
|  | LS8759 | *h^+^ leu1-32 ura4-D18 his3-D1 nbs1-c60∆::13myc-kanMX6* |
|  | LS9212^a^ | *h^-^ leu1-32 ura4-D18 his3-D1 rad3-kd∆::ura4^+^ nbs1-c60∆::13myc-kanMX6 // pREP41H-rad3 (P_nmt1(p41)_::rad3^+^; his3^+^)* |
|  | LS9217^a^ | *h^+^ leu1-32 ura4-D18 ade6-M210 his3-D1 rad3∆::LEU2 nbs1-c60∆::13myc-kanMX6 // pREP41H-rad3 (P_nmt1(p41)_::rad3^+^; his3^+^)* |
|  |  |  |
| 2D | TN2411 | *h^-^ leu1-32 ura4-D18 his3-D1* |
|  | LS8733 | *h^+^ leu1-32 ura4-D18 ade6-704 his3-D1 9myc::tel1^+^* |
|  | LS8730 | *h^+^ leu1-32 ura4-D18 ade6-704 his3-D1 9myc::tel1^+^ rad3-kd∆::ura4^+^* |
|  | LS8726 | *h^+^ leu1-32 ura4-D18 ade6-704 his3-D1 9myc::tel1^+^ rad3∆::LEU2* |
|  | LS9030 | *h^+^ leu1-32 ura4-D18 ade6-704 his3-D1 9myc::tel1^+^ nbs1-c60∆::GFP-kanMX6* |
|  | LS9193^a^ | *h^+^ leu1-32 ura4-D18 ade6-M216 or -704 his3-D1 9myc::tel1^+^ rad3-kd∆::ura4^+^ nbs1-c60∆::GFP-kanMX6 // pREP41H-rad3 (**P_nmt1(p41)_::rad3^+^; his3^+^)* |
|  | LS9196^a^ | *h^+^ leu1-32 ura4-D18 ade6-M210 or -704 his3-D1 9myc::tel1^+^ rad3∆::LEU2 nbs1-c60∆::GFP-kanMX6 // pREP41H-rad3 (P_nmt1(p41)_::rad3^+^; his3^+^)* |
|  | LS9033 | *h^+^ leu1-32 ura4-D18 ade6-704 his3-D1 9myc::tel1^+^ nbs1∆::kanMX6* |
|  | LS9220^a^ | *h^+^ leu1-32 ura4-D18 ade6-M216 or -704 his3-D1 9myc::tel1^+^ rad3-kd∆::ura4^+^ nbs1∆::kanMX6 // pREP41H-rad3 (P_nmt1(p41)_::rad3^+^; his3^+^)* |
|  | LS9223^a^ | *h^+^ leu1-32 ura4-D18 ade6-M210 or -704 his3-D1 9myc::tel1^+^ rad3∆::LEU2 nbs1∆::kanMX6 // pREP41H-rad3 (P_nmt1(p41)_::rad3^+^; his3^+^)* |
|  |  |  |
| 3A | TN2411 | *h^-^ leu1-32 ura4-D18 his3-D1* |
|  | LS7938 | *h^-^ leu1-32 ura4-D18 his3-D1 9myc::rad3^+^* |
|  | LS9019 | *h^+^ leu1-32 ura4-D18 his3-D1 9myc::rad3^+^ nbs1-c60∆::GFP-kanMX6* |
|  | LS9022 | *h^+^ leu1-32 ura4-D18 his3-D1 9myc::rad3^+^ nbs1∆::kanMX* |
|  | LS8640 | *h^-^ leu1-32 ura4-D18 his3-D1 9myc::rad3-kd∆::kanMX4* |
|  | LS9190^a^ | *h^+^ leu1-32 ura4-D18 his3-D1 9myc::rad3-kd∆::kanMX4 nbs1-c60∆::GFP-kanMX6 // pREP41H-rad3 (P_nmt1(p41)_::rad3^+^; his3^+^)* |
|  | LS9870^a^ | *h^-^ leu1-32 ura4-D18 his3-D1 9myc::rad3-kd∆::kanMX4 nbs1∆::natMX // pREP41H-rad3 (P_nmt1(p41)_::rad3^+^; his3^+^)* |
|  |  |  |
| 3B | TN2411 | *h^-^ leu1-32 ura4-D18 his3-D1* |
|  | TN5599 | *h^-^ leu1-32 ura4-D18 his3-D1 rad11^+^-5FLAG::kanMX* |
|  | TN6324 | *h^-^ leu1-32 ura4-D18 his3-D1 rad3-kd∆::ura4+ rad11^+^-5FLAG::kanMX* |
|  | LS9858 | *h^-^ leu1-32 ura4-D18 ade6-M216 his3-D1 rad11^+^-5FLAG::kanMX nbs1-c60∆::GFP-kanMX6* |
|  | LS9863^a^ | *h^+^ leu1-32 ura4-D18 his3-D1 rad3-kd∆::ura4+ rad11^+^-5FLAG::kanMX nbs1-c60∆::GFP-kanMX6 // pREP41H-rad3 (P_nmt1(p41)_::rad3^+^; his3^+^*) |
|  | LS9860 | *h^+^ leu1-32 ura4-D18 his3-D1 rad11^+^-5FLAG::kanMX nbs1∆::natMX* |
|  | LS9864^a^ | *h^+^ leu1-32 ura4-D18 his3-D1 rad3-kd∆::ura4+ rad11^+^-5FLAG::kanMX nbs1∆::kanMX6 // pREP41H-rad3 (P_nmt1(p41)_::rad3^+^; his3^+^* |
|  | TN8069^a^ | *h^-^ leu1-32 ura4-D18 ade6-M210 his3-D1 tel1∆::LEU2* *rad3∆::LEU2 rad11^+^-5FLAG::kanMX // pREP41H-rad3 (P_nmt1(p41)_::rad3^+^; his3^+^)* |
|  |  |  |
| 4A | LS8825^b^ | *h^-^ leu1-32 ura4-D18 his3-D1* (+B1) |
|  | LS8833^b^ | *h^-^ leu1-32 ura4-D18 his3-D1* (-B1) |
|  | LS8818^b^ | *h^-^ leu1-32 ura4-D18 his3-D1 kanMX6-P_nmt1(p1)_-3HA::tel1^+^* (+B1) |
|  | LS8826^b^ | *h^-^ leu1-32 ura4-D18 his3-D1 kanMX6-P_nmt1(p1)_-3HA::tel1^+^* (-B1*)* |
|  | LS8822^b^ | *h^-^ leu1-32 ura4-D18 his3-D1 kanMX6-P_nmt1(p1)_-3HA::tel1^+^ rad3-kd∆::ura4^+^* (+B1) |
|  | LS8830^b^ | *h^-^ leu1-32 ura4-D18 his3-D1 kanMX6-P_nmt1(p1)_-3HA::tel1^+^ rad3-kd∆::ura4^+^* (-B1) |
|  | LS8820^b^ | *h^-^ leu1-32 ura4-D18 his3-D1 kanMX6-P_nmt1(p1)_-3HA::tel1^+^ rad3∆::LEU2* (+B1) |
|  | LS8828^b^ | *h^-^ leu1-32 ura4-D18 his3-D1 kanMX6-P_nmt1(p1)_-3HA::tel1^+^ rad3∆::LEU2* (-B1) |
|  |  |  |
| 4B | LS8819^b^ | *h^+^ leu1-32 ura4-D18 his3-D1 kanMX6-P_nmt1(p1)_-3HA::tel1^+^ nbs1-c60∆::GFP-kanMX6* (+B1) |
|  | LS8827^b^ | *h^+^ leu1-32 ura4-D18 his3-D1 kanMX6-P_nmt1(p1)_-3HA::tel1^+^ nbs1-c60∆::GFP-kanMX6* (-B1) |
|  | LS9717^b^ | *h^+^ leu1-32 ura4-D18 his3-D1 kanMX6-P_nmt1(p1)_-3HA::tel1^+^ nbs1∆::natMX* (+B1) |
|  | LS9718^b^ | *h^+^ leu1-32 ura4-D18 his3-D1 kanMX6-P_nmt1(p1)_-3HA::tel1^+^ nbs1∆::natMX* (-B1) |
|  | LS8823^b^ | *h^+^ leu1-32 ura4-D18 his3-D1 kanMX6-P_nmt1(p1)_-3HA::tel1^+^ rad3-kd∆::ura4^+^ nbs1-c60∆::GFP-kanMX6* (+B1) |
|  | LS8831^b^ | *h^+^ leu1-32 ura4-D18 his3-D1 kanMX6-P_nmt1(p1)_-3HA::tel1^+^ rad3-kd∆::ura4^+^ nbs1-c60∆::GFP-kanMX6* (-B1) |
|  |  |  |
| 4B,C | LS9689 | *h^+^ leu1-32 ura4-D18 his3-D1 kanMX6-P_nmt1(p1)_-3HA::tel1^+^ rad3-kd∆::ura4^+^ nbs1∆::natMX* (+B1) |
|  | LS9690 | *h^+^ leu1-32 ura4-D18 his3-D1 kanMX6-P_nmt1(p1)_-3HA::tel1^+^ rad3-kd∆::ura4^+^ nbs1∆::natMX* (-B1) |
|  | LS8821 | *h^+^ leu1-32 ura4-D18 his3-D1 kanMX6-P_nmt1(p1)_-3HA::tel1^+^ rad3∆::LEU2 nbs1-c60∆::GFP-kanMX6* (+B1) |
|  | LS8829 | *h^+^ leu1-32 ura4-D18 his3-D1 kanMX6-P_nmt1(p1)_-3HA::tel1^+^ rad3∆::LEU2 nbs1-c60∆::GFP-kanMX6* (-B1) |
|  | LS9694 | *h^+^ leu1-32 ura4-D18 his3-D1 kanMX6-P_nmt1(p1)_-3HA::tel1^+^ rad3∆::LEU2 nbs1∆::natMX* (+B1) |
|  | LS9739 | *h^+^ leu1-32 ura4-D18 his3-D1 kanMX6-P_nmt1(p1)_-3HA::tel1^+^ rad3∆::LEU2 nbs1∆::natMX* (-B1) |
|  | LS8824 | *h^-^ leu1-32 ura4-D18 his3-D1 kanMX6-P_nmt1(p1)_-3HA::tel1^+^ rad26∆::ura4^+^ nbs1-c60∆::GFP-kanMX6* (+B1) |
|  | LS8832 | *h^-^ leu1-32 ura4-D18 his3-D1 kanMX6-P_nmt1(p1)_-3HA::tel1^+^ rad26∆::ura4^+^ nbs1-c60∆::GFP-kanMX6* (-B1) |
|  | LS9691 | *h^+^ leu1-32 ura4-D18 his3-D1 kanMX6-P_nmt1(p1)_-3HA::tel1^+^ rad26∆::ura4^+^ nbs1∆::natMX* (+B1) |
|  | LS9692 | *h^+^ leu1-32 ura4-D18 his3-D1 kanMX6-P_nmt1(p1)_-3HA::tel1^+^ rad26∆::ura4^+^ nbs1∆::natMX* (-B1) |
|  |  |  |
| 4D | TN2411 | *h^-^ leu1-32 ura4-D18 his3-D1* |
|  | LS4999 | *h^-^ leu1-32 ura4-D18 his3-D1 kanMX6-P_nmt1(p1)_-3HA::tel1^+^* |
|  | LS8639 | *h^-^ leu1-32 ura4-D18 his3-D1 kanMX6-P_nmt1(p1)_-3HA::tel1^+^ rad3-kd∆::ura4^+^* |
|  | LS6747 | *h^-^ leu1-32 ura4-D18 his3-D1 kanMX6-P_nmt1(p1)_-3HA::tel1^+^ rad3∆::LEU2* |
|  | LS6130 | *h^+^ leu1-32 ura4-D18 his3-D1 kanMX6-P_nmt1(p1)_-3HA::tel1^+^ nbs1-c60∆::GFP-kanMX6* |
|  | LS8762 | *h^+^ leu1-32 ura4-D18 his3-D1 kanMX6-P_nmt1(p1)_-3HA::tel1^+^ rad3-kd∆::ura4^+^ nbs1-c60∆::GFP-kanMX6* |
|  | LS6380 | *h^+^ leu1-32 ura4-D18 his3-D1 kanMX6-P_nmt1(p1)_-3HA::tel1^+^ rad3∆::LEU2 nbs1-c60∆::GFP-kanMX6* |
|  |  |  |
| 5A | LS8945^b^ | *h^-^ leu1-32 ura4-294::_Pnmt1(p1)_-3HA-rad3^+^-ura4^+^ his3-D1 rad26^+^::hphMX6* (+B1) |
|  | LS8949^b^ | *h^-^ leu1-32 ura4-294::_Pnmt1(p1)_-3HA-rad3^+^-ura4^+^ his3-D1 rad26^+^::hphMX6* (-B1) |
|  | LS8948^b^ | *h^-^ leu1-32 ura4-294::_Pnmt1(p1)_-3HA-rad3^+^-ura4^+^ rad26∆::ura4^+^* (+B1) |
|  | LS8952^b^ | *h^-^ leu1-32 ura4-294::_Pnmt1(p1)_-3HA-rad3^+^-ura4^+^ rad26∆::ura4^+^* (-B1) |
|  |  |  |
| 5B | LS9744^b^ | *h^+^ leu1-32 ura4-294::P_nmt1(p1)_-3HA-rad3^+^-ura4^+^ his3-D1 rad26∆::ura4^+^ nbs1-c60∆::GFP-kanMX6* (+B1) |
|  | LS9874^b^ | *h^+^ leu1-32 ura4-294::P_nmt1(p1)_-3HA-rad3^+^-ura4^+^ his3-D1 rad26∆::ura4^+^ nbs1-c60∆::GFP-kanMX6* (-B1) |
|  | LS9873^b^ | *h^+^ leu1-32 ura4-294::P_nmt1(p1)_-3HA-rad3^+^-ura4^+^ rad26∆::ura4^+^ nbs1-c60∆::GFP-kanMX6* (+B1) |
|  | LS9875^b^ | *h^+^ leu1-32 ura4-294::P_nmt1(p1)_-3HA-rad3^+^-ura4^+^ rad26∆::ura4^+^ nbs1-c60∆::GFP-kanMX6* (-B1) |
|  | LS9876^b^ | *h^+^ leu1-32 ura4-294::P_nmt1(p1)_-3HA-rad3^+^-ura4^+^ his3-D1 rad26∆::ura4^+^ nbs1∆::kanMX6* (+B1) |
|  | LS10095^b^ | *h^+^ leu1-32 ura4-294::P_nmt1(p1)_-3HA-rad3^+^-ura4^+^ his3-D1 rad26∆::ura4^+^ nbs1∆::kanMX6* (-B1) |
|  | LS9907^b^ | *h^+^ leu1-32 ura4-294::P_nmt1(p1)_-3HA-rad3^+^-ura4^+^ his3-D1 rad26∆::ura4^+^ nbs1∆::kanMX6* (+B1) |
|  | LS9908^b^ | *h^+^ leu1-32 ura4-294::P_nmt1(p1)_-3HA-rad3^+^-ura4^+^ his3-D1 rad26∆::ura4^+^ nbs1∆::kanMX6* (-B1) |
|  | LS9633^b^ | *h^-^ leu1-32 ura4-294::P_nmt1(p1)_-3HA-rad3^+^-ura4^+^ ade6-M210 tel1∆::LEU2* *rad26∆::ura4^+^* (+B1) |
|  | LS9634^b^ | *h^-^ leu1-32 ura4-294::P_nmt1(p1)_-3HA-rad3^+^-ura4^+^ ade6-M210 tel1∆::LEU2* *rad26∆::ura4^+^* (-B1) |
|  | LS9740^b^ | *h^+^ leu1-32 ura4-294::P_nmt1(p1)_-3HA-rad3^+^-ura4^+^ ade6-M210 tel1∆::LEU2* *rad26∆::ura4^+^* (+B1) |
|  | LS9742^b^ | *h^+^ leu1-32 ura4-294::P_nmt1(p1)_-3HA-rad3^+^-ura4^+^ ade6-M210 tel1∆::LEU2* *rad26∆::ura4^+^* (-B1) |
|  |  |  |
| 5C | TN2411 | *h^-^ leu1-32 ura4-D18 his3-D1* |
|  | LS8783 | *h^-^ leu1-32 ura4-294::_Pnmt1(p1)_-3HA-rad3^+^-ura4^+^ his3-D1 rad26^+^::hphMX6* |
|  | TN1789 | *h^-^ leu1-32 ura4-294::_Pnmt1(p1)_-3HA-rad3^+^-ura4^+^ rad26∆::ura4^+^* |
|  |  |  |
| 5D | LS9569 | *h^+^ leu1-32 ura4-294::P_nmt1(p1)_-3HA-rad3^+^-ura4^+^ his3-D1 rad26∆::ura4^+^ nbs1-c60∆::GFP-kanMX6* |
|  | LS10093 | *h^+^ leu1-32 ura4-294::P_nmt1(p1)_-3HA-rad3^+^-ura4^+^ ade6-704 his3-D1 9myc::tel1^+^ rad26∆::ura4^+^ nbs1-c60∆::GFP-kanMX6* |
|  |  |  |
| S1A | TN2411 | *h^-^ leu1-32 ura4-D18 his3-D1* |
|  | TN846 | *h^-^ leu1-32 ura4-D18 ade6-M216 his3-D1 rad3-kd∆::ura4^+^* |
|  | TN1329 | *h^+^ leu1-32 ura4-D18 ade6-M216 his3-D1 rad3∆::LEU2* |
|  | TN854 | *h^-^ leu1-32 ura4-D18 ade6-M216 his3-D1 rad26∆::ura4^+^* |
|  | TN1567 | *h^-^ leu1-32 ura4-D18 ade6-M216 his3-D1 rad3∆::LEU2 rad26∆::ura4^+^* |
|  |  |  |
| S1B | TN2411  TN1678  TN1374  TN4377  LS9203^a^  LS9208^a^ | *h^-^ leu1-32 ura4-D18 his3-D1*  *h^-^ leu1-32 ura4-D18 ade6-M216 his3-D1 rad3-kd∆::kanMX4*  *h^-^ leu1-32 ura4-D18 ade6-M210 his3-D1 rad3∆::LEU2*  *h^+^ leu1-32 ura4-D18 ade6-M216 his3-D1 nbs1-c60∆::GFP-kanMX6*  *h^+^ leu1-32 ura4-D18 ade6-M216 his3-D1 rad3-kd∆::ura4^+^ nbs1-c60∆::GFP-kanMX6 // pREP41H-rad3 (P_nmt1(p41)_::rad3^+^; his3^+^)*  *h^-^ leu1-32 ura4-D18 ade6-M210 or -M216 his3-D1 rad3∆::LEU2 nbs1-c60∆::GFP-kanMX6 // pREP41H-rad3 (P_nmt1(p41)_::rad3^+^; his3^+^)* |
| S1C,D | TN2411  TN1678  TN1374  TN854 | *h^-^ leu1-32 ura4-D18 his3-D1*  *h^-^ leu1-32 ura4-D18 ade6-M216 his3-D1 rad3-kd∆::kanMX4*  *h^-^ leu1-32 ura4-D18 ade6-M210 his3-D1 rad3∆::LEU2*  *h^-^ leu1-32 ura4-D18 ade6-M216 his3-D1 rad26∆::ura4^+^* |
| S1E,F | LS9203^a^ | *h^+^ leu1-32 ura4-D18 ade6-M216 his3-D1 rad3-kd∆::ura4^+^ nbs1-c60∆::GFP-kanMX6 // pREP41H-rad3 (P_nmt1(p41)_::rad3^+^; his3^+^)* |
|  | LS9208^a^ | *h^-^ leu1-32 ura4-D18 ade6-M210 or -M216 his3-D1 rad3∆::LEU2 nbs1-c60∆::GFP-kanMX6 // pREP41H-rad3 (P_nmt1(p41)_::rad3^+^; his3^+^)* |
|  | LS9226^a^ | *h^+^ leu1-32 ura4-D18 his3-D1 rad3-kd∆::ura4+ nbs1∆::kanMX6 // pREP41H-rad3 (P_nmt1(p41)_::rad3^+^; his3^+^)* |
|  | LS9230^a^ | *h^+^ leu1-32 ura4-D18 his3-D1 rad3∆::LEU2 nbs1∆::kanMX6 // pREP41H-rad3 (P_nmt1(p41)_::rad3^+^; his3^+^)* |

^a^Strains that have lost the Rad3 plasmid (*pREP41H-rad3*) were used in experiments.

^b^Cultured in minimal media supplemented with (+B1) or without (-B1) thiamine for many generations to repress or induce *nmt1^+^* promoter controlled proteins, respectively.
