# Supplementary material for: A Kinase-Independent Role for the Rad3ATR-Rad26ATRIP Complex in Recruitment of Tel1ATM to Telomeres in Fission Yeast
Source: PLoS Genet. 2010 Feb 5;6(2):e1000839. doi: 10.1371/journal.pgen.1000839 (PMC2816689; doi:10.1371/journal.pgen.1000839)
Supplement: Table S2 — DNA primers used in strain construction. (0.06 MB DOC) [file pgen.1000839.s003.doc]

| **Table S2.** DNA primers used in strain construction. | | |
| --- | --- | --- |
| **Strain** | **Primer Name** | **Primer Sequence (5’ to 3’)** |
| *rad3Δ::LEU2* (*rad3Δ*) | rad3-LEUT^a^ | TCAAGACTTTGAACGCGCGTGTTGCGTTTTAAAAAGGCCTTTTTTTGAATTGAATCAATGGTTTGATATAGTgaggagaacttctagtatatccacatac |
|  | rad3-LEUB^a^ | CTAGAAATAAGCAGCCCAACCAATGTACATTTCTACCAGGTTTTTTGGGTTGACAGCAGATTTGATCAATTCtacgtcgtaaggccgtttctgacagagt |
|  |  |  |
| *myc-rad3* | rad3-nmt-up-T | AGCATCGCTCGATACTTAGTG |
|  | rad3-9myc-B^b^ | ATTAAGGGTTGTCGACCTGCACATactatatcaaaccattgattcaat |
|  | rad3-9myc-T^b^ | AGTGGCCTATGCGGCCGCagccaacacgcaaaaaggaaagct |
|  | rad3-nmt-TAP-B | TGTTTAAAAGCTAGAAGACCAAC |
|  |  |  |
| *nbs1-c60∆-myc* | nbs1-T12 | attagaaggattcagttgcgccatgttaa |
|  | nbs1dC60-myc-B^c^ | TTAATTAACCCGGGGATCCGAtcatcttggtcaaacacttc |
|  | nbs1-tag-T4 | CGGATCCCCGGGTTAATTAACGGT |
|  | kan-B5^d^ | ggcggcgttagtatcgaatcgac |
|  | kan-T4^d^ | ACTGCTGTCGATTCGATACTAACGCCGCC |
|  | nbs1-B13 | cccttcaattgttaatatctgcatgaaaactt |
|  |  |  |
| *myc-tel1* | tel1-B4 | GCTTCATTTAGCGACGCTGTTTCGTTTATC |
|  | tel1-9myc-B^b^ | ATTAAGGGTTGTCGACCTGCACATagtattacatcgagtaaaagttat |
|  | tel1-9myc-T^b^ | AGTGGCCTATGCGGCCGCacttctctaaatgacatcgttaat |
|  | tel1-B7 | TTTTCAATAGCTTGACAACAATAGGAAACT |
|  |  |  |
| *nmt-HA-tel1* | tel1-nmtT^e^ | TTCCAAAAGTTGGAGACGCGTAACCTTTTACTCAATTATCAGTTTACGCCAAATATGTTAATATATTAATGAAGAAAAGAgaattcgagctcgtttaaac |
|  | nmt-3HAtel1-B^e^ | CGAAGATTTTGTAGAGCATCTGATCGTGTTTTGATTTTTGATGAAGACAACTTATTAACGATGTCATTTAGAGAAGTgcactgagcagcgtaatctg |
|  |  |  |
| *nbs1∆::natMX* | BAM98^d^ | GCGACATGGAGGCCCAGAATACC |
|  | kan-B5^d^ | ggcggcgttagtatcgaatcgac |

^a^Underlined sequences anneal to *S. cerevisiae* *LEU2* gene.

^b^Underlined sequences anneal to pOM20 plasmid [Gauss *et al.* (2005) Yeast **22**: 1-12].

^c^Underlined sequences anneal to *13myc* or *3HA* regions.

^d^Underlined sequences anneal to *kanMX6* or *natMX*.

^e^Underlined sequences anneal to pFA6a-kanMX6-p3nmt-3HA plasmid [Bähler *et al.* (1998) Yeast **14:** 943-951].
