# Supplementary material for: A Kinase-Independent Role for the Rad3ATR-Rad26ATRIP Complex in Recruitment of Tel1ATM to Telomeres in Fission Yeast
Source: PLoS Genet. 2010 Feb 5;6(2):e1000839. doi: 10.1371/journal.pgen.1000839 (PMC2816689; doi:10.1371/journal.pgen.1000839)
Supplement: Table S3 — Plasmids used in this study. (0.04 MB DOC) [file pgen.1000839.s004.doc]

| **Table S3.** Plasmids used in this study. | | |
| --- | --- | --- |
| **Plasmid** | **Genes** | **Description** |
| pAS1-rad3 | *TRP1; GAL4-DNA-BD::rad3^+^* | Yeast 2-hybrid plasmid |
| pAS1-rad3-kd∆ | *TRP1; GAL4-DNA-BD::rad3-kd∆* | Yeast 2-hybrid plasmid |
| pAS1-rad3(1-191) | *TRP1; GAL4-DNA-BD::rad3(1-191)* | Yeast 2-hybrid plasmid |
|  |  |  |
| pGAD424-rad26 | *LEU2; GAL4-AD::rad26^+^* | Yeast 2-hybrid plasmid |
|  |  |  |
| pREP41H-rad3 | *his3^+^; P_nmt1(p41)_::rad3^+^* | Rad3 plasmid used to maintain telomeres |
